# Structure-Dependent Activity of Natural GABA(A) Receptor Modulators

**DOI:** 10.3390/molecules23071512

**Published:** 2018-06-22

**Authors:** Serhat Sezai Çiçek

**Affiliations:** Department of Pharmaceutical Biology, Kiel University, Gutenbergstraße 76, 24118 Kiel, Germany; scicek@pharmazie.uni-kiel.de; Tel.: +49-431-880-1077

**Keywords:** γ-aminobutyric acid, natural product, plant origin, benzodiazepine binding site, anxiolytic, sedative, anesthetic, anticonvulsant

## Abstract

GABA(A) receptors are ligand-gated ion channels consisting of five subunits from eight subfamilies, each assembled in four hydrophobic transmembrane domains. This pentameric structure not only allows different receptor binding sites, but also various types of ligands, such as orthosteric agonists and antagonists, positive and negative allosteric modulators, as well as second-order modulators and non-competitive channel blockers. A fact, that is also displayed by the variety of chemical structures found for both, synthetic as well as nature-derived GABA(A)-receptor modulators. This review covers the literature for natural GABA(A)-receptor modulators until the end of 2017 and discusses their structure-activity relationship.

## 1. Introduction

GABA (γ-aminobutyric acid) type A receptors are members of the ligand-gated ion-channel superfamily, consisting of four hydrophobic transmembrane domains (TM1–TM4) [1]. Around the pore, five subunits form eight subunit subfamilies (α, β, γ, δ, ε, θ, π and ρ) assemble to form a heteropentameric chloride (Cl^−^)-permeable channel [2]. Despite the extensive heterogeneity of the GABA(A) receptor subunits, most GABA(A) receptors expressed in the brain consist of two α subunits, two β subunits and one γ subunit [1]. Each receptor subunit also contains a large intracellular domain between TM3 and TM4, which is the site for various protein interactions as well as for various post-translational modifications that modulate receptor activity [1]. Its endogenous ligand is GABA, the major inhibitory neurotransmitter in the central nervous system, which upon activation selectively conducts Cl^−^ through its pore, resulting in hyperpolarization of the neuron [3]. This causes an inhibitory effect on the neurotransmission by diminishing the chance of a successful action potential occurring [3]. The active site of the GABA(A) receptor is the binding site for GABA and several drugs such as muscimol, gaboxadol and bicuculline [3]. Additionally, the receptor contains several different allosteric binding sites, which modulate the activity of the receptor indirectly and are the targets of various other drugs, such as benzodiazepines, barbiturates, ethanol, picrotoxin, general anaesthetics and neuroactive steroids [4,5,6,7].

Due to the structural heterogeneity of GABA(A) receptors and the number of binding sites, natural (and synthetic) compounds with a variety of chemical structures were discovered to possess GABA(A) receptor modulating properties and depending on the mode of action, the affected binding site and the compounds’ affinity, to exhibit diverse pharmacological effects. Investigation of these effects was accomplished using different in vitro and in vivo models. Of the in vitro studies, about half of the reports result from radioligand binding assays, where compounds were tested for their ability to displace a ligand from its respective binding site. Possible binding sites to be studied with these assays are the GABA/muscimol-, the benzodiazepine- and the *tert*-butylbicyclophosphorothionate (TBPS)/picrotoxin-binding site [8]. Other in vitro models to determine GABAergic activity are electrophysiological studies, such as voltage clamp techniques. In these systems, compounds can be investigated for their stimulation or inhibition of chloride currents on recombinant GABA(A) receptors expressed in different cell types, allowing to measure receptors composed of different subunits. Additional in vivo studies helped to understand the correlation of subtype modulation and the respective pharmacological effects, such as anxiolytic, sedative and anticonvulsive properties.

Several animal models are described to assess anxiolytic potential, which evaluate either conditioned or unconditioned responses [9]. Conditioned responses are measured in the Vogel conflict (VC) and the four-plate-test (FP), where mild electroshocks are used to punish drinking or exploratory behavior, respectively [9,10]. Evaluation of unconditioned responses is accomplished using the elevated plus maze (EPM), the open field (OF), the holeboard (HB) or the light/dark exploration test (LD), where the aversion of rodents to elevated, open or brightly lit areas is determined [10]. Other animal models to measure effects on anxiety and/or stress are the marble burying test (MB), the forced swim test (FS), the tail suspension test (TS) and stress-induced hyperthermia (SIH) [10,11]. The open field test can also be used to assess locomotor activity (LMA) of the animals, determining the number of crossings, rears and grooms as well as the total distance travelled [12]. Other models to measure sedative/hypnotic effects are pentobarbital-induced sleeping (PIS) or ethyl ether-induced sleeping (EIS), where an increase in sleep latency and sleep duration are measured. To assess anticonvulsant properties, animals are treated with either electroshocks (MES), pentetrazol (PTZ) or picrotoxin (PTX) in order to provoke seizures [13]. Parameters to be measured are the number and intensity of convulsions, seizure latency and time to death. Additional animal models discussed in this review are the rotarod (RR) and the horizontal wire test (HW), where compounds are tested for their myorelaxant properties as well as the tail-flick (TF) response and the tail immersion test (TI), which are used to measure analgesic effects [14,15,16,17].

Meanwhile, several pharmacological effects can be attributed to the modulation of specific α-subunits [2,18]. Sedative and hypnotic properties, for example, are mediated via GABA(A) receptors containing α1-subunits, whereas positive modulation of GABA(A) receptors with α2 and/or α3-subunits displays anxiolytic effects. Moreover, α5-subunits play a role in learning and memory. Benzodiazepines, common therapeuticals for the treatment of anxiety, insomnia and convulsions, are known to act on α1, α2, α3 and α5 subunits, which is the reason for some of their side-effects, such as muscle relaxation or anterograde amnesia [18,19]. Other disadvantages are the development of tolerance and dependence. Therefore, the discovery of new and safer medicines, with, e.g., strong anxiolytic but weak sedative potential, is urgently warranted. In recent decades, various reports have been made on natural products with GABAergic activity and, as already mentioned, different kinds of methods have been used to describe the effects. Hence, the aim of this review was to collect the existing data and make the obtained results as comparable as possible, thus facilitating the discussion of structure activity relationships.

## 2. Method

A literature search was carried out using the Web of Science citation indexing service and the terms “GABA receptor” and “gamma-aminobutyric acid receptor” in combination with the words “natural”, “naturally occurring” or “plant-derived”, resulting in 795 publications. Additionally, the terms “GABA receptor” and “gamma-aminobutyric acid receptor” were searched alone and the results were reduced to the field of plant science, giving 244 hits. The publications were reviewed by title, abstract and text and reduced to those dealing with GABA type A receptors. Furthermore, studies of extracts (without identifying active single molecules) were excluded. Data of the remaining publications was collected and categorized by the pharmacological methods used (voltage clamp techniques, radioligand binding assays and in vivo studies) and the chemical structures of the active molecules. Chemical structures were divided into compound classes (alkaloids, alkanes, phenols and terpenes) and, depending on the number compounds, into several subclasses, thereby defining the structure of this review. Compound names (mostly trivial names) and configurations were taken “as is” from the original publications and species names of the natural sources used in the studies were checked using “The Plant List” or “Index Fungorum”, respectively [20,21]. If not specified otherwise, doses administered in the in vivo studies were intraperitoneal and animals tested were either mice or rats. Diazepam was generally used as reference compound.

The data discussed in this review is additionally summarized in four tables at the end of this review. Data from electrophysiological recordings is divided into antagonists (Table 1) and agonists (Table 2). Here, compound number, source, assay and expressed subtype combination are given, as well as EC_50_ values and maximal stimulation for agonists and IC_50_ values and percent inhibition for antagonist, respectively. If results of different receptor subtypes were available, those measured with α_1_β_2_γ_2_ are presented. If no maximal stimulation was given in the original publications, the highest obtained value and concentration is presented instead. Table 3 shows data from radioligand binding assays. As above, compound number, source, IC_50_ values and maximal stimulation are given, as well as Ki values and the applied radioligand. Compounds are ordered by the studied binding site. In Table 4, data from the in vivo studies is depicted. Here, either the lowest active dose is presented and, if no doses are given, + or − is shown if significant results were (+) or were not observed (−). In the tables as well as in the main text, only results with a *p* value below 5% (*p* < 0.05) were considered.

## 3. Natural GABA(A) Receptor Modulators

### 3.1. Alkaloids

**Figure 1:**
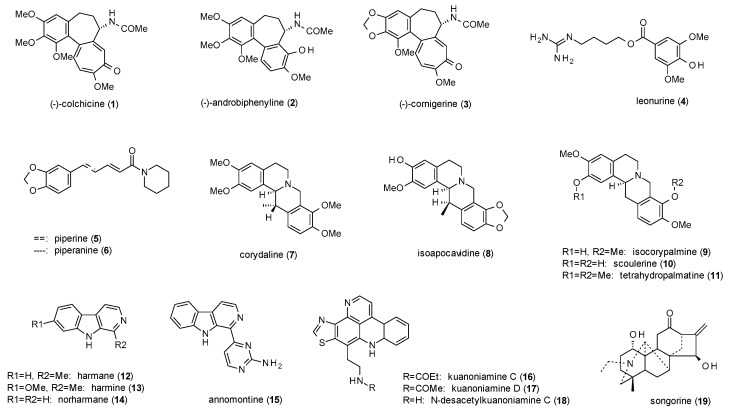
Chemical structures of alkaloids with reported GABA(A)-receptor modulating activity.

A total of 19 alkaloids (including four protoalkaloids) have been reported to possess GABA(A)-modulating activity (Figure 1).

Alali et al. isolated (−)-colchicine (**1**), (−)-androbiphenyline (**2**) and (−)-cornigerine (**3**) along with six other colchicinoids from *Colchicum brachyphyllum*, which meanwhile is classified as *Colchicum szovitsii* ssp. *brachyphyllum* [22]. Of the nine isolated compounds, **2** and **3** acted as weak partial agonists in a radioligand binding assay using [^35^S]TBPS and [^3^H]flunitrazepam. The two compounds exhibited about 25% of the stimulation of 10 µM allopregnanolone, while (−)-colchicine showed no activity. This finding is in accordance with a previous study of Bueno et al., which identified (−)-colchicine as a competitive antagonist of GABA, decreasing the GABAergic responses to 40% of the control level [23]. 

Another protoalkaloid with reported GABAergic activity is leonurine (**4**) from *Leonurus japonicus*, an East Asian herbal remedy for the treatment of anxiety, depression, nervousness, and insomnia [24]. In a radioligand assay with [^3^H]gabazine and [^3^H]flumazenil the compound showed IC_50_ values of 15 µg/mL and 123 µg/mL, respectively.

Piperidine-alkaloids piperine (**5**) and piperanine (**6**) were investigated in *Xenopus laevis* oocytes by Zaugg et al. [25]. At a concentration of 100 µM and in the presence of GABA, the compounds induced potentiation of chloride currents of 226% and 138%, respectively. The maximum stimulation observed was 302% (**5**) and 187% (**6**), and EC_50_ values were calculated as 52 and 56 µM, respectively. As the effects were not antagonized by flumazenil, the observed allosteric modulation must be independent from the benzodiazepine binding site.

Halbsguth et al. isolated four protoberberine type 1 and five protoberberine type 2 alkaloids (**7**–**11**) from the rhizomes of *Corydalis cava* [26]. While type 1 alkaloids elicited no effect in a radioligand assay with [^3^H]BMC (bicuculline methochloride), type 2 alkaloids enhanced BMC binding by 21–49% at concentrations of 0.1 µM (**7**, **8**,**11**) and 0.01 µM (**9**,**10**), indicating the necessary saturation of both heterocycles. Scoulerine (**10**) was additionally investigated on the increase of Muscimol-Alexa binding, increasing the amount of hindered mobility of the receptor ligand complex comparable to midazolam. In vivo studies on tetrahydropalmatine (**11**), which was isolated from *Corydalis yanshuo*, showed an increased number of open arm entries and time spent in the open arms at doses from 0.5 to 10 mg/kg p.o. in the EPM and a decrease of time spent in open arms and closed arm entries at 30 and 50 mg/kg p.o. [27]. At a dose of 50 mg/kg p.o. the compound also caused a decrease in head dips in the holeboard test and led to performance deficits in the horizontal wire test.

Farzin et Mansouri investigated the antidepressant-like effect of β-carbolines harmane (**12**), harmine (**13**) and norharmane (**14**) in the forced swim test on mice [28]. Harmane and harmine dose-dependently reduced the immobility time from 5 to 15 mg/kg, while norharmane was already effective at a dose of 2.5 mg/kg. Flumazenil (5 mg/kg) completely antagonized the effect, suggesting the antidepressant effect to be triggered via the benzodiazepine binding site.

Another β-carboline with GABAergic activity, annomontine (**15**), was isolated from *Annona purpurea* [29]. In the EPM test, the compound increased the time spent in the open arms and the open arm entries at 10 and 30 mg/kg, but not the total arm entries. Flumazenil at a dose of 3 mg/kg antagonized the effect and thus confirmed the above-mentioned effect of β-carbolines on the benzodiazepine binding site. However, in the open field test, annomontine showed no differences on the number of lines crossed and the number of rearings compared to vehicle and diazepam. Neither did the investigation of locomotor activity in the rotarod test and the pentobarbital-induced sleeping test show any significant results.

Eder et al. reported the isolation of three pyridoacridine alkaloids, kuanoniamine C (**16**), D (**17**) and N-desacetylkuanoniamine (**18**) from a Micronesian sponge of the genus *Oceanapia* [30]. In a radioligand assay all three compounds showed moderate affinity to the benzodiazepine binding site, inhibiting the binding of [^3^H]diazepam by 30 to 39% at a test concentration of 25 µM each.

The last alkaloid to be discussed in this review is songorine (**19**), a diterpene alkaloid which was isolated from *Aconitum leucostomum* and found to be a non-competitive GABA(A) receptor antagonist, with an IC_50_ value of 19.6 µM [31]. Additional radioligand studies using [^3^H]muscimol resulted in an IC_50_ value of 7.06 µM and a Kd value of 6.31 nM. Nesterova et al. investigated the anxiolytic effect of songorine (isolated from *Aconitum barbatum*) using the Vogel conflict test [32]. The compound at a dose of 0.25 mg/kg intragastrically led to an increase in punished drinks without producing any sedative effect.

Regarding the subtype specifity of the presented alkaloids, only β-carbolines (**12**–**14**) and picroacridine alkaloids (**15**–**18**) can be attributed to a specific binding site, which in this case is the benzodiazepine binding site. For piperidine alkaloids (**5**,**6**) and protoberberine alkaloids (**7**–**11**) this binding site can at least be excluded, as can the the GABA/muscimol binding site for songorine (**19**). The three colchicinoids (**1**–**3**) seem to exhibit unspecific binding with no (**1**) or weak (**2** and **3**) stimulation on both the benzodiazepine as well as the TBPS/bicuculline binding site. The more interesting fact is that colchicine (**1**) acts as receptor antagonist, whereas androbiphenyline (**2**) and cornigerine (**3**) are both partial agonists. Also, the fourth protoalkaloid, leonurine (**4**), shows binding to different sites, with weak affinities to both, the GABA/muscimol and the benzodiazepine binding site. Another finding is that only protoberberine type 2 alkaloids were able to modulate GABA(A) receptors, whereas unsaturated type 1 alkaloids exhibited no effects.

### 3.2. Alkanes

**Figure 2:**
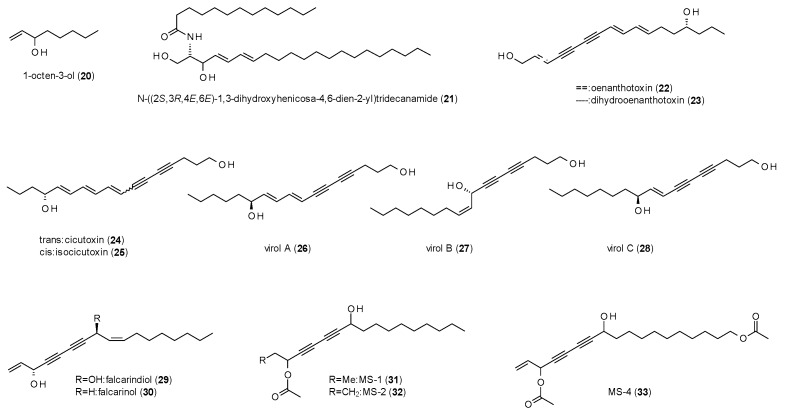
Chemical structures of alkanes with reported GABA(A)-receptor modulating activity.

In this section, 14 compounds will be described, of which twelve belong to the class of polyacetylenes (Figure 2).

The first compound, 1-octen-3-ol (**20**), was part of a study on the GABA(A) receptor modulation of odor substances, which will be described in the section on terpenes [33]. Anyway, at a concentration of 300 µM and 1 µM GABA, 1-octen-3-ol exhibited a stimulation of 295 ± 50%.

The next compound, *N*-[(2*S*,3*R*,4*E*,6*E*)-1,3-dihydroxyhenicosa-4,6-dien-2-yl]tridecanamide (**21**), is a ceramide, isolated from the Red Sea soft coral *Sarcophytum auritum* [34]. The compound was reported to increase the time spent in the open arms in the EPM test and the time spent in the light are in the light/dark test as well as the time to death after injection of pentylenetetrazol (PTZ). All actions were antagonized by bicuculline (0.1 mg/kg), proving the effects to be mediated via a GABAergic pathway. However, no information is given on the doses of **21** used in the different assays.

Oenanthotoxin (**22**) and dihydrooenanthotoxin (**23**), two polyacetylenes isolated form *Oenanthe fistulosa* exhibited strong inhibitory effects on GABA(A) receptors, with IC_50_ values of 0.835 µM and 1.29 µM, respectively [35]. The authors conclude that this block of GABAergic responses is the molecular rationale for the symptoms of poisoning from water-dropwort (*Oenanthe crocata*) and a possible explanation for the facial muscular contractions, also known as *risus sardonicus*. A follow-up study on the mechanisms of action of oenanthotoxin found the compound to allosterically modulate GABA-binding and non-competitively block the ion channel at the same time, nearby abolishing receptor desensitization [36].

Uwai et al. isolated five polyacetylenes, cicutoxin (**24)**, isocicutoxin (**25**), virol A (**26**), B (**27**) and C (**28**) from water hemlock (*Cicuta virosa*) [37]. A radioligand binding study using [^3^H]EBOB resulted in IC_50_ values of 0.541 µM (**24**), 2.01 µM (**25**), 1.15 µM (**26**), 6.01 µM (**27**) and 7.87 µM (**28**). Further studies with semisynthetic derivatives of the compounds revealed that both, the terminal as well as the allylic hydroxy-group are both important for the toxicity of the compounds.

In a study on falcarindiol (**29**) isolated from *Oenanthe crocata*, the compound was found to potently modulate GABAergic currents at low micromolar concentrations [38]. A finding that was approved by a follow-up study by Czyzewska et al., who isolated falcarindiol along with falcarinol (**30**) from *Bunium bulbocastanum* [39]. Both compounds were found to enhance GABA-induced ion currents at low micromolar concentrations (1 µM for falcarindiol and 0.1 µM for falcarinol), but rather block GABAergic activity at higher concentrations. The authors assume that the low-dose effect is the reason for the sedative but not convulsive effect in animals, while the higher concentrations in herbivorous insects act insecticidal.

The last three polyacetylenes with reported GABA(A)-modulating activity were isolated from *Cussonia zimmermannii* and named MS-1 (**31**), MS-2 (**32**) and MS-4 (**33**) [40]. The three compounds showed a stimulation of chloride currents from 110 to 440% with EC_50_ values ranging from 0.6 to 3.5 µM.

Of the 14 compounds discussed in this section, twelve belong to the class of polyacetylenes (**22**–**33**), a compound class with interesting GABAergic activities. Altogether, they show efficacies and/or affinities in the low micromolar range but differ strongly in terms of toxicity. According to Uwai et al. the toxicity is caused by two structural features, the allylic and the terminal hydroxy-group, which are both present in the five (most) toxic natural compounds of his study (**24**–**28**). Regarding the data of the other reported polyacetylenes, it can further be concluded that the terminal hydroxy-group is more important for the toxicity, as both oenanthotoxins (**22**,**23**) lack the allylic hydroxy-group but are strongly poisonous. In contrast, falcarinol (**30**) and falcarindiol (**29**), which have an allylic but no terminal hydroxy-group, show reduced toxicity. The last three polyacetylenes (**31**–**33**) have neither of the two “toxic features”, and are also not reported to exhibit inhibitory activity, which is in accordance with this theory. However, as MS-4 (**33**) has a terminal acetyloxy-group, it would be interesting to study if hydrolisation led to GABA(A) receptor antagonism.

### 3.3. Phenols

#### 3.3.1. Flavones

**Figure 3:**
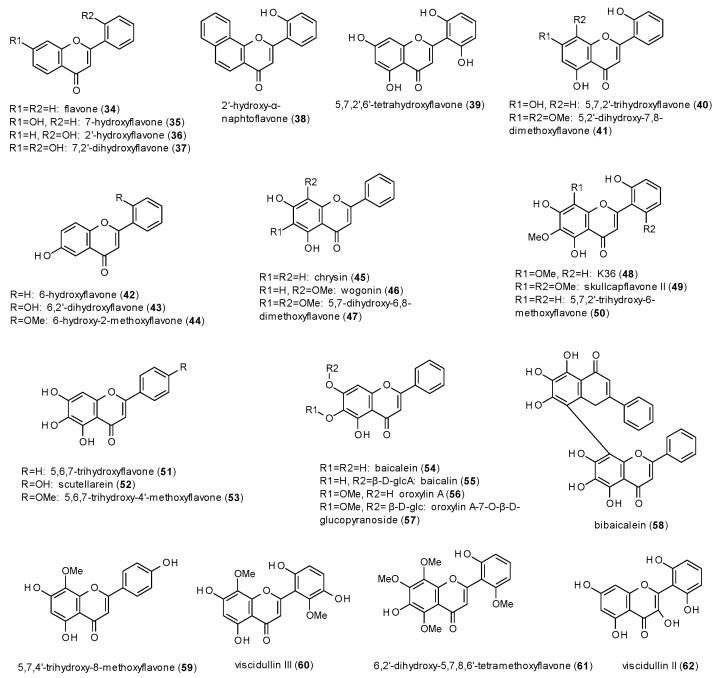
Chemical structures of flavones from *Scutellaria baicalensis* with reported GABA(A)-receptor modulating activity.

Most natural products with GABAergic activity have been reported from the class of flavones, with 29 compounds isolated from *Scutellaria baicalensis* alone (Figure 3) [41,42].

A radioligand binding assay using [^3^H]flunitrazepam revealed sub-micromolar Ki or IC_50_ values for eleven of the isolated compounds (**36**–**38**,**40**,**41**,**43**,**45**,**47**–**50**). Compound **48** even showed affinity to the benzodiazepine site at a nanomolar level (6.1 ± 0.1 nM), which was similar to that of diazepam (6.4 ± 0.2 nM). Due to the number of structurally very similar compounds tested in the two studies, some findings regarding the structure-activity relationship could be drawn:

- Hydroxylation in position 5 and 7 or in position 6 leads to increased receptor affinity.
- Methoxylation in position 6 or 8 also raises affinity of the compounds, even more when occurring in both positions.
- The affinity furthermore increases by hydroxylation in position 2’, while methoxylation in the same position leads to a loss of affinity.

Wang et al. additionally tested four flavanones, which altogether showed a much lower activity than the respective flavones and, except for **87** (see next subsection), produced no considerable effect.

The major constituents of *Scutellaria baicalensis* were also part of other in vitro and in vivo studies. Baicalein (**54**) and its glucuronide baicalin (**55**), for example, were tested for their anxiolytic potential in the Vogel conflict test, where both compounds were found to increase the number of accepted shocks at doses of 10 mg/kg [43]. Baicalin was also found to increase number of open arm entries and time spent in the open arms in an EPM test after central (i.c.v.) application of 5.4 and 54 ng of the compound [44]. At the same doses baicalein also increased ethyl ether-induced sleeping time but had no effect on PTZ-induced seizures. Thus, the authors concluded that the anxiolytic-like and sedative effects are mediated through GABA(A) receptors, but not via the benzodiazepine binding site. In a study on the sleep-wake regulation of baicalin, the compound at doses of 42.7 and 85.3 ng led to a decrease of slow wave sleep during the first 2 h in the light period and to an increase of slow wave sleep of hours 8 to 12 in the dark period [45]. In the latter period, a dose of 85.3 ng also caused an increase of rapid eye movement.

Oroxylin A (**56**), was tested for its effect on chloride currents in single cortical neurons and thereby found to allosterically inhibit GABA-mediated receptor modulation [46], thus explaining the outcome of an earlier in vivo study by Huen et al., where the compound did not show any significant results but antagonizing the effects provoked by diazepam [47]. Interestingly, oroxylin A only differs from baicalein by the presence of a methoxy-group in position 6.

Wogonin (**46**) was investigated on the potentiation of GABA-induced chloride currents, where it exhibited a stimulation of 57% at a concentration of 30 µM in the presence of 1 µM GABA [48]. The calculated EC_50_ value was 3 µM. In the same study, wogonin showed an increase in head dips and time spent head dipping in the holeboard test, as well as an increase in total arm entries and open arm entries at the EPM test. The effects were observed at doses of 7.5, 15 and 30 mg/kg p.o. No sedative or myorelaxant effects were observed in the locomotor activity test or the horizontal wire test at doses up to 30 mg/kg p.o. Neither did intraperitoneal injection of 0.2 to 10 mg/kg lead to a decrease in locomotor activity in a study on the anticonvulsant effect [49]. In this study, wogonin reduced the intensity of maximal electroshock (MES)-induced seizures and PTZ (70 mg/kg)-induced seizures at doses of 5 and 10 mg/kg. The higher doses also reduced the intensity seizures induced by 100 mg/kg PTZ.

The last flavone from *Scutellaria baicalensis* to be discussed is chrysin (**45**), though the compound has been the target of earlier studies, where it was isolated from *Passiflora caerulea* [50,51,52]. Medina et al. found the compound to be anticonvulsive, when applied i.c.v., but not when applied i.p. [50]. Wolfman et al. observed an increase in locomotor activity at doses from 0.6 to 1 mg/kg and an increase of open arm entries and time spent in the open arms at a dose of 1 mg/kg, an effect that was blocked by flumazenil [51]. The horizontal wire test showed a decreased percentage of animals grasping the horizontal wire, while in the holeboard test an increase in time spent head-dipping at 3 mg/kg was observed, but no sedative effects at doses of 3 and 6 mg/kg. Zhai et al. reported a decrease in tail withdrawal latencies after oral administration of 25, 50, 75 and 100 mg/kg chrysin in the tail immersion test [52]. As the effect was antagonised by flumazenil (0.75 and 1 mg/kg), bicuculline (2 and 4 mg/kg) and picrotoxin (1 and 2 mg/kg) the authors conclude that the hyperalgesic effect is associated with GABA(A) receptors. A study on the effect of chrysin and other flavones on memory tasks showed by Salgueiro et al. did not reveal significant results for any of the tested compounds [53]. An in vitro study of chrysin on GABA-induced ion currents showed a slight inhibition (9.7% at 10 µM), which reached its maximum (12.1%) at 30 µM [54].

**Figure 4:**
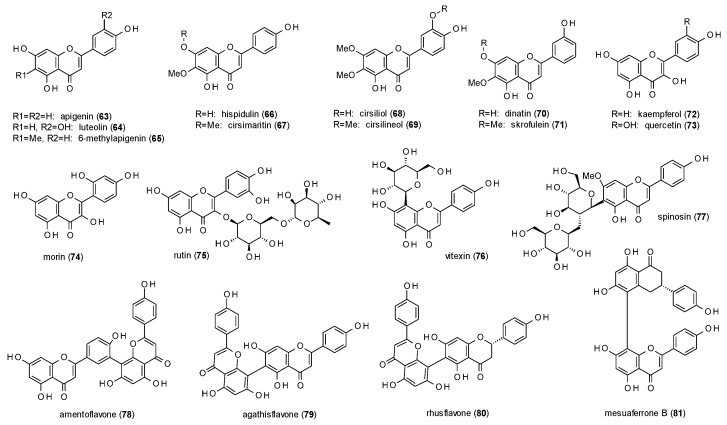
Chemical structures of other flavones with reported GABA(A)-receptor modulating activity.

In the same study, apigenin (**63**), quercetin (**73**) and morin (**74**) were identified as GABA inhibitors, with maximal inhibitions of 50.6, 84.5 and 38.4%, respectively (Figure 4). Similar results for apigenin were obtained by Avallone et al., who tested inhibition of chloride currents in cerebellar granule cells and found the effect to be antagonized by flumazenil [55]. Campbell et al. and Kavvadias et al. described this effect to be allosterically mediated and reported IC_50_ values of 8 and 6.9 µM, respectively [56,57].

Due to its high abundance in the plant kingdom, apigenin was subject of various studies. Viola et al. were testing the affinity to the benzodiazepine binding site using [^3^H]flunitrazepam and determined a Ki value of 4 µM [58], while other studies were using [^3^H]flumazenil and calculated IC_50_ values of 10 to 30 µM and a Ki value of 9 µM [57,59,60]. Viola et al. also tested the anxiolytic-like behaviour in the EPM test and found an increased percentage of open arm entries and time spent in the open arms but no differences in total arm entries at a dose of 3 mg/kg, while sedative effects were only observed at doses of 30 and 100 mg/kg, respectively [58]. The sedative effects of apigenin at higher doses (25 and 50 mg/kg) were confirmed by Avallone et al. but no anxiolytic-like effects in the EPM test were noticed at doses of 0.5 to 10 mg/kg [55]. Apigenin also decreased the latency of picrotoxin-induced seizures at 25 and 50 mg/kg, but had no effect on death rate and death latency. Neither did the compound exhibit myorelaxant activity at a dose of 50 mg/kg. Gazola et al. investigated the sedative effect of apigenin isolated from *Passiflora quadrangularis* and measured an increase of sleep duration at a dose of 0.6 mg/kg p.o. [61].

Another very common flavonoid with GABA-inhibiting properties is luteolin (**64**), which was found to reduce stimulation of chloride currents to 66% of the control level at a concentration of 50 µM [62]. The IC_50_ value was calculated with 6.62 µM. 

Wasowski et al. isolated 6-methylapigenin (**65**) from *Valeriana wallichii* (now classified as *Valeriana jatamansi*) and subjected the compound to a radioligand assay using [^35^S]TBPS [63]. The obtained Ki value was 0.50 µM. In a follow-up study 6-methylapigenin was tested in the EPM test, where it increased the percentage of open arm entries and time spent in the open arms at a dose of 1 mg/kg [64]. The compound additionally potentiated the sleep-enhancing properties of hesperidin, which was also part of this study and will discussed later in this review.

Hispidulin (**66**) and cirsimaritin (**67**) were isolated from *Salvia officinalis* and studied for their affinity to the benzodiazepine binding site using [^3^H]flumazenil [65]. Even though both compounds only differ by methylation of the 7-hydroxy-group their IC_50_ values vary strongly with 1.3 µM calculated for hispidulin and 350 µM for cirsimaritin. Hispidulin was further subjected to electrophysiological studies in *Xenopus laevis* oocytes showing a maximal potentiation of 47% at a concentration of 10 µM [57]. The authors additionally investigated the effect of hispidulin on Mongolian gerbils. After seven days of treatment with 10 mg/kg p.o. per day milder seizures were observed and the number of animals suffering from seizures was significantly reduced. Salah et al. isolated hispidulin together with cirsilineol (**68**) from *Artemisia herba-alba* and found IC_50_ values of 8 and 100 µM in a radioligand binding assay with [^3^H]flumazenil [66].

Cirsiliol (**69**), a derivative of cirsilineol, was isolated by Viola et al. from *Salvia guaranitica*, which is meanwhile classified as *Salvia coerulea* [67]. The compound was measured against [^3^H]zolpidem (Ki of 20 µM) and [^3^H]flunitrazepam (Ki value of 200 µM) and investigated in the pentobarbital-induced sleeping test, where it showed hypnotic effects at doses from 2 to 10 mg/kg. Moreover, cirsiliol showed produced no myorelaxant or anticonvulsant effects at doses of 30 and 10 mg/kg, respectively.

Shen et al. isolated dinatin (**70**) and skrofulein (**71**) from *Artemisia herba-alba* and tested the compounds in a radioligand assay using [*methyl*-^3^H]diazepam and obtained IC_50_ values of 1.3 and 22.7 µM, respectively [68].

A bioguided fractionation procedure of an aqueous extract of the leaves of *Apocynum venetum* using the EPM test led to the identification of kaempferol (**72**) as one of the active principles [69]. Examination of the isolated substance led to an increase in the time spent in the open arms at doses from 0.02 to 1.0 mg/kg after oral administration. Vissiennon et al. also found the compound to be anxiolytic in the EPM test at a dose of 1 mg/kg p.o., but not after intraperitoneal application of the same dose [70]. The same effect was observed for quercetin (**73**), which increased open arm entries and the time spent in the open arms already at a dose of 0.5 mg/kg p.o. The authors thereupon investigated the metabolites of these two compounds and found para-hydroxy-phenylacetic acid to be active in the same way after i.p. administration. After gut sterilization of the mice used in the study with enrofloxaxin and oral administration of kaempferol and quercetin no anxiolytic-like effect was observed. Thus, the authors concluded that both compounds act as prodrugs, which need to be activated by the intestinal flora.

Rutin (**75**), a flavonol diglycoside, was investigated for its effect on PTZ-induced seizures [71]. I.c.v. injection of 5 µL of 50 nM solution prolonged the latency of general tonic-clonic seizures, whereas injection of a 150 nM solution also increased the latency of minimal clonic seizures. The C-glycoside vitexin (**76**) was also effective on both parameters at concentrations of 100 and 200 nM [72]. Pre-treatment with flumazenil (5 nM) abolished the anticonvulsant effects.

Spinosin (**77**) was isolated from *Ziziphus spinosa* and tested for its anxiolytic-like properties [73]. In the EPM test, spinosin increased the open arm entries and the time spent in the open arms at doses of 2.5 and 5 mg/kg. In the light/dark test, an increase in transitions and time spent in the light area was measured at a dose of 5 mg/kg. At the same dose, an increased number of entries into the centre was observed in the open field test. All effects were antagonized by flumazenil and WAY-100635, indicating that the effects are modulated by both, GABA(A) as well as 5-HT_1A_ receptors.

Amentoflavone (**78**), a biflavone, was isolated from *Hypericum perforatum* and measured against [^3^H]flumazenil in a radioligand binding assay, giving an IC_50_ value of 14.9 nM [74]. In the same study, the compound was found to pass the blood-brain barrier in vitro by passive diffusion.

Amentoflavone, together with agathisflavone (**79**), was also isolated from *Rhus pyroides*, which is now classified as *Searsia pyroides* [58]. Both compounds were studied in a radioligand binding assay with [^3^H]flumazenil and showed Ki values of 37 and 28 nM, respectively.

Phytochemical investigations on *Rhus parviflora*, a taxon which is still unresolved, led to the isolation of agathisflavone, rhusflavone (**80**) and mesuaferrone B (**81**), which were tested against [^35^S]TBPS [75]. The compounds showed Ki values of 91, 45 and 280 nM, and IC_50_ values of 149, 73 and 455 nM, respectively. Rhusflavone additionally decreased sleep latency in the pentobarbital-induced sleeping test at doses of 12.5, 25 and 50 mg/kg and prolonged sleep duration at doses of 25 and 50 mg/kg. Pre-administration of flumazenil (8 mg/kg) reduced sleep duration and increased sleep latency at a dose of 50 mg/kg rhusflavone.

The structure-activity findings observed for the flavones isolated from *Scutellaria baicalensis* are confirmed when looking at the data of the other reported flavones. The effect of the 5,7-dihydroxy-susbtitution also becomes obvious when comparing hispidulin (**66**) to cirsimaritin (**67**) or dinatin (**70**) to skrofulein (**71**), where methylation of the hydroxy-group in position 7 leads to higher Ki or IC_50_ values. The IC_50_ values of apigenin (**63**) and hispidulin (**66**) in the [^3^H]flumazenil binding assay, on the other hand, demonstrate increased affinity through methoxylation in position 6. The third finding, in which the hydroxylation in position 2’ increases affinity, cannot be confirmed directly, but considering the nanomolar IC_50_ value of amentoflavone (**78**) in the [^3^H]flunitrazepam binding assay, it seems that the 7-hydroxy-group of the second monomer has a similar impact on the receptor affinity. Regarding the affinity of flavones to the TBPS/picrotoxin binding site, all four tested flavones (**65**,**79**–**81**) showed sub-micromolar IC_50_ and Ki values (against [^35^S]TBPS). In terms of receptor modulation, most flavones are reported to exhibit negative allosteric modulation, apart from wogonin (**46**) and hispidulin (**66**), which show weak potentiation of GABA-induced ion currents. In vivo studies revealed anxiolytic properties for apigenin (**63**), kaempferol (**72**) and quercetin (**73**). Thereby, the latter two compounds were found to be prodrugs, with para-hydroxyphenylacetic acid as the active metabolite.

#### 3.3.2. Flavanes

**Figure 5:**
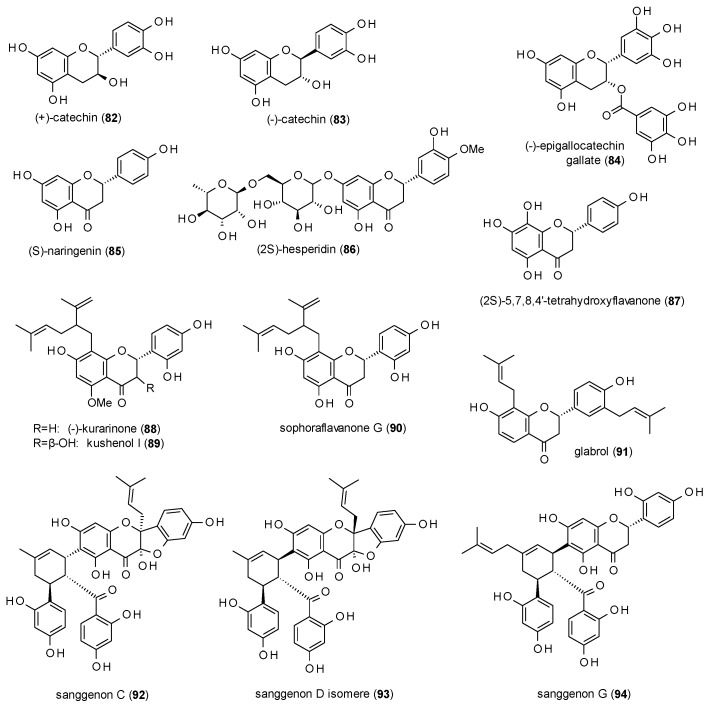
Chemical structures of flavanes with reported GABA(A)-receptor modulating activity.

In contrast to the high number of flavones, the group of GABA(A) receptor modulating flavanes only consists of 14 compounds (Figure 5).

(+)- and (−)-catechin (**82** and **83**) have been tested for their effect on [^3^H]γ-hydroxybutyric acid (GHB) binding and on the binding of [^3^H]NCS-382, a GHB antagonist in order to find new lead compounds for the GHB high-affinity site of the GABA(A) receptor [76]. (+)-catechin enhanced [^3^H]NCS-382 binding to 163% and decreased [3H]GHB-binding to 54%, whereas (−)-catechin was less active. (+)-catechin was subsequently investigated on GABA(A) receptors composed of α_4_β_4_δ subunits expressed in *Xenopus* oocytes, where it exhibited a stimulation of 68% at a concentration of 100 µM.

(−)-epigallocatechin (**84**) at a concentration of 100 µM inhibited responses to 40 µM GABA up to 90.6% and that of 20 µM GABA up to 97% with IC_50_ values of 14.7 and 8.7 µM, respectively [55]. Furthermore, the compound reduced sleeping latency and prolonged sleep duration in the pentobarbital-induced sleeping test at doses from 5 to 20 mg/kg p.o. [77].

(S)-naringenin (**85**) was isolated from an ethanolic leaf extract of *Mentha aquatica* and tested against [^3^H]flumazenil, showing an IC_50_ value of 26 mM [78].

(2S)-hesperidin (**86**) at a dose of 4 mg/kg was found to decrease locomotor activity and reduce the exploration of holes as well as the number of rearings in the holeboard test [63]. The compound at the same dose increased the thiopental-induced sleeping time. The same levels of sleeping time were achieved at a dose of 2 mg/kg in combination with 1 mg/kg of apigenin (**63**).

(2*S*)-5,7,8,4′-tetrahydroxyflavone (**87**) is one of the few flavanones from *Scutellaria baicalensis* isolated by Wang et al. with an IC_50_ value of 21.4 µM against [^3^H]flunitrazepam [41].

Yang et al. isolated three 8-lavandulyl flavanones with GABAergic activity, (−)-kurarinone (**88**), kushenol I (**89**) and sophoraflavanone G (**90**) from the Chinese herbal drug Kushen (*Sophora flavescens*) and investigated the potentiation of GABA-induced chloride currents in *Xenopus* oocytes [79]. At a concentration of 10 µM the compounds exhibited a potentiation of 719.7, 216.5 and 211.6%, with EC_50_ values of 4.0, 5.0 and 15.0 µM, respectively.

Glabrol (**91**), a prenylated flavanone from liquorice, was tested against [^3^H]flumazenil with an Ki value of 1.63 µM [80].

Kim et al. isolated three Diels-Alder type adducts, sanggenon C (**92**), sanggenon D isomer (**93**) and sanggenon G (**94**) from the root bark of *Morus alba* [81]. All three compounds enhanced GABA-induced chloride currents by more than 700% (at 100 µM), with EC_50_ values from 13.4 to 16.7 µM.

As mentioned in the last subsection, *Scutellaria baicalensis* also yielded several flavanones, which altogether showed much lower receptor affinities than their corresponding flavones. However, other studies resulted in the isolation of prenylated flavanones, which did exhibit noteworthy effects. Two 8-lavandulyl-flavanones (**88** and **90**), for example, caused a potentiation of GABA-induced chloride currents of around 600%. The third one (**89**), an 8-lavandulyl-flavanonol, was significantly less active. Three other prenylated flavanones (**92**–**94**) even exhibited stimulations of more than 700%. Glabrol (**91**) was the only prenylated flavanone investigated for receptor affinity and showed a Ki of 1.63 against [^3^H]flumazenil, which was far lower than any tested unprenylated flavanone, indicating that prenylation can lead to both, higher receptor affinity as well as pronounced potentiation of chloride currents.

#### 3.3.3. Isoflavonoids and Chalcones

**Figure 6:**
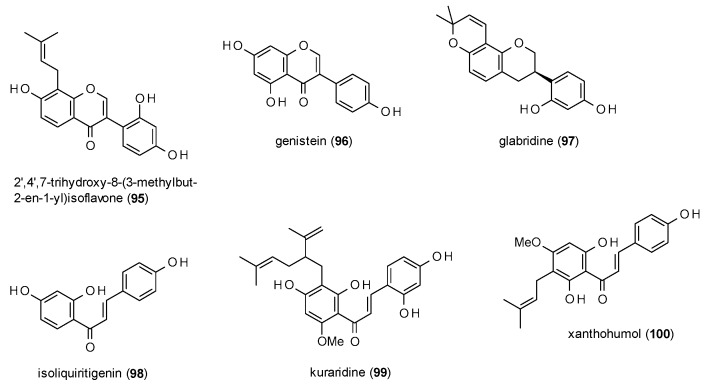
Chemical structures of isoflavonoids and chalcones with reported GABA(A)-receptor modulating activity.

Due to the limited number of reports, isoflavones (including one isoflavane) and chalcones will be discussed in one subsection (Figure 6).

2′,4′,7-trihydroxy-8-(3-methylbut-2-en-1-yl)isoflavone (**95**), which was isolated from *Adenocarpus cinncinatus*, exhibited a potentiation of GABA-induced chloride currents of 552.73% at a concentration of 500 µM, with an EC_50_ value of 2.8 µM [82].

Genistein (**96**) at a concentration of 100 µM inhibited responses to 40 µM GABA up to 51% with an IC_50_ value 29.2 µM [55].

Glabridine (**97**), another compound from liquorice, showed remarkable effects on GABA(A) receptors expressed in dorsal raphe neurons [83]. The compound enhanced GABA-induced chloride currents by 135% at a concentration of only 30 nM, with a maximal potentiation of 581% at a concentration of 3 µM.

Cho et al. investigated isoliquiritigenin (**98**) with a patch-clamp technique on dorsal raphe neurons, where the compound enhanced GABA-induced currents by 151% at a concentration of 10 µM [84]. Further characterization of the compound resulted in a Ki value of 0.453 µM in a radioligand assay with [^3^H]flunitrazepam as well as a decrease of sleep latency and an increase in sleep duration at doses of 25 and 50 mg/kg p.o.

Kuraridine (**99**), a lavandulyl chalcone from *Sophora flavescens* potentiated GABA-induced chloride currents by 719.7% at a concentration of 10 µM with a maximum stimulation of 891.5% [79]. The EC_50_ value was calculated with 4.0 µM.

Xanthohumol (**100**), a chalcone found in hops (*Humulus lupulus*), enhanced Mu-Alexa binding from 5.2 nM to 6.1 nM at a concentration of 75 nM [85].

The results for isoflavonoids and chalcones are in accordance with the findings of the last two subsections: the isoflavone genistein (**96**) inhibits chloride currents in the same manner as its flavone counterpart apigenin (**63**) and the chalcone isoliquiritigenin (**98**) blocks [^3^H]flunitrazepam binding as efficiently as many of the before mentioned flavones. In addition, also here, the prenylated forms exhibit pronounced potentiation from over 500% (**95**,**97**) to almost 900% (**99**).

#### 3.3.4. Phenylpropanes, Kavalactones and Lignans

**Figure 7:**
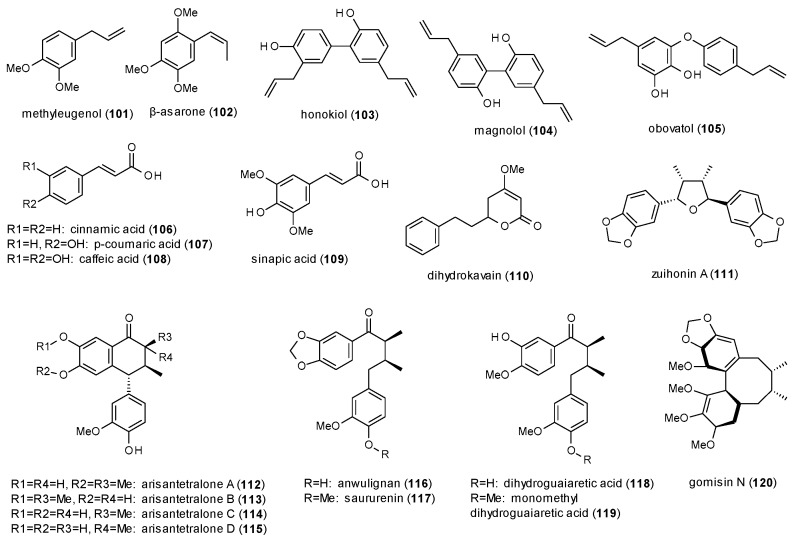
Chemical structures of phenylpropanes, kavalactones and lignans with reported GABA(A)-receptor modulating activity.

This subsection contains simple and condensed phenylpropanes as well as one kavalactone (Figure 7).

Methyleugenol (**101**) was investigated on GABA(A) receptors expressed in HEK-293T cells and identified as direct agonist with a stimulation of 290% [86].

β-asarone (**102**), isolated from *Acorus calamus*, showed a remarkable maximal potentiation of GABA-induced chloride currents in a *Xenopus* oocyte assay of 1200% at a concentration of 500 µM [87]. However, the calculated EC_50_ value of 171.5 µM was rather high.

Another compound with a pronounced stimulation of GABA-induced chloride currents is honokiol (**103**), a dimeric phenylpropane from *Magnolia officinalis* [88]. Honokiol exhibited a maximal stimulation (1315% at 300 µM) comparable to that of β-asarone, but showed a much lower EC_50_ value (36.2 µM). The effect was even higher at GABA(A) receptors composed of α_3_β_2_ subunits, reaching a maximal stimulation of more than 2000%. In an earlier study, honokiol was already found to increase the time spent in the open arms in an EPM test at a dose of 20 mg/kg p.o. and that the effect was blocked by flumazenil (0.3 mg/kg s.c.) and bicuculline (0.1 mg/kg s.c.) [89]. Another study reported that the compound increased the sleeping time in the pentobarbital-induced sleeping test at doses of 0.1 and 0.2 mg/kg p.o., but had no effect on the sleep latency [90]. Alexeev et al. tested honokiol and magnolol (**104**) on different populations of neuronal and recombinant GABA(A) receptors, finding that both compounds enhanced phasic and tonic GABAergic neurotransmission and that the compounds were active on all subtypes [91]. The authors conclude that the modulation of synaptic as well as extra-synaptic populations of GABA(A) receptors could lead to significant side effects and risk of drug interactions.

Ma et al. isolated obovatol (**105**) from *Magnolia obovata* and reported the compound enhance the pentobarbital-induced sleeping time at doses of 0.05, 0.1 and 0.2 mg/kg p.o. [92]. Furthermore, obovatol significantly increased the chloride influx into the cultured cerebral granule cells and increased the expression of α-, β- and γ-subunits.

Scheepens et al. investigated cinnamic acid (**106**), p-coumaric acid (**107**) and caffeic acid (**108**) on GABA(A) receptors expressed in HEK-293T cells [93]. All three compounds activated the receptor with EC_50_ values of 10.1, 6.9 and 10.5 µM, respectively. Further studies on p-coumaric acid revealed that the compound also exhibited anxiolytic effects in the EPM test at 30 and 90 mg/kg and that the effect was blocked by picrotoxin.

Electrophysiological experiments on sinapic acid (**109**) showed a stimulation of GABA-induced chloride currents of 158% at a concentration of 10 µM and an EC_50_ value of 42.58 nM [94]. At a dose of 4 mg/kg p.o. the compound was also found to increase the number of head dips in the holeboard test and the time spent in the open arms in the EPM test. Both bicuculline (5 mg/kg) and flumazenil (10 mg/kg) reversed the effect.

Dihydrokavain (**110**) is the only kavalactone with reported GABA(A) receptor modulation, enhancing chloride currents by 31.5% at a concentration of 300 µM and showing an EC_50_ value of 93 µM [95].

Zaugg et al. isolated zuihonin A (**111**), arisantetralones A–D (**112**–**115**) and four acylic lignans (**116**–**119**) with GABAergic activity from *Kadsura longipedunculata* [96]. In a *Xenopus* oocyte assay highest stimulation of chloride currents was observed for arisantetralone B (885.8%) and dihydroguaiaretic acid (793.4%), while the lowest EC_50_ values were found for saururenin (12.8 µM) and zuihonin A (21.8 µM).

Gomisin N (**120**) was isolated from *Schisandra chinensis* and investigated on the potentiation of hypnotic effects in the pentobarbital-induced sleeping test [97]. The compound reduced sleep latency and prolonged sleep duration at concentrations of 15, 30 and 45 mg/kg. Co-application of flumazenil (8 mg/kg) reduced sleep duration but did not increase sleep latency.

In this subsection, only the lignans allow a structure-activity discussion, as nine compounds were investigated in the same test system (**111**–**119**). Of these nine compounds, those two exhibited the highest potentiation, which were substituted with one hydroxy- and one methoxy-group on both aromatic rings (**113**,**118**). If only one ring had this substitution pattern, a methylenedioxo-group was superior to two hydroxy-groups in the second ring. The substitution pattern with one hydroxy-group next to a methoxy-group is reminiscent of the increased receptor affinity of flavones, where highest affinity was observed for a 5,7-dihydroxy-6,8-dimethoxy-substitution pattern.

#### 3.3.5. Coumarins

**Figure 8:**
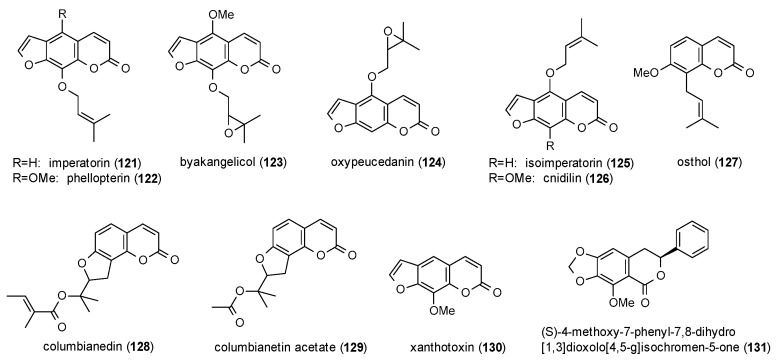
Chemical structures of coumarins with reported GABA(A)-receptor modulating activity.

Eleven coumarins were reported to possess GABAergic activity, of which seven belong to the subclass of linear furanocoumarins (Figure 8).

First reports were made by Bergendorff et al., who isolated imperatorin (**121**), phellopterin (**122**) and byakangelicol (**123**) from the roots of *Angelica dahurica* and tested their affinity to the benzodiazepine binding site using [^3^H]diazepam [98]. IC_50_ values of the three compounds were 8.0, 0.36 and 12 µM, respectively. 

Imperatorin and phellopterin were also investigated on GABA(A) receptors expressed in *Xenopus* oocytes along with oxypeucedanin (**124**), isoimperatorin (**125**) and osthol (**127**) [99]. Osthol and oxypeucedanin showed the lowest EC_50_ values (14 and 25 µM) and were therefore investigated for their stimulation of GABA-induced chloride currents. Here, oxypeucedanin at a concentration of 100 µM exhibited a potentiation of 550%, while osthol was significantly less active (124%).

Osthol was also isolated from *Angelica pubescens* together with imperatorin, cnidilin (**126**), columbianedin (**128**) and columbianetin acetate (**129**), but only osthol showed a noteworthy potentiation of GABA-induced chloride currents [100]. In a study on the protection against MES-induced seizures osthol exhibited protective effects at doses from 259 to 631 mg/kg [101]. Similar results were obtained for imperatorin showing protection at a dose of 300 mg/kg [102]. Imperatorin as well as isoimperatorin showed a decrease in head dip latency and an increased number of rearings at a dose of 5 mg/kg p.o. in the holeboard test [103]. At the same dose, both compounds caused an increase in the number of open arm entries and time spent in the open arms in the EPM test, as well as a decrease in closed arm entries and time spent in the closed arms. In the light/dark test both compounds increased the time spent in the light area and decreased the time spent in the dark area at a dose of 5 mg/kg p.o., with isoimperatorin additionally increasing the number of crossings.

Xanthotoxin (**130**), a furanocoumarin isolated from *Pastinaca sativa*, was tested for its protection against MES-induced convulsions [102,104,105]. Luszczki et al. found the compound to be protective at a dose of 300 mg/kg [102]. Skalicka-Wozniak et al. calculated median protective doses against hind limb tonic extension from 219 to 252 mg/kg [104], and Zagaja et al. found the compound to be protective from hind limb tonic extension at a dose of 150 mg/kg [105]. Another finding of the latter study was that xanthotoxin enhanced the anticonvulsive effect of carbamazepin and valproate at 100 mg/kg.

The only GABA(A) receptor-modulating isocoumarin (**131**) was isolated from *Haloxylon scoparium*, but the compound showed only moderate stimulation of chloride currents (144.6% at a concentration of 500 µM) and an EC_50_ value of 140.2 µM [106].

Regarding the structure-activity relationship of coumarins, Singhuber et al. found the prenyl residue essential for positive GABA(A) receptor modulation, with oxyprenylated oxypeucedanin (**124**) exhibiting the highest potentiation. Additional findings were that both, bulkier moieties as well as the lack of the C5-side chain lead to a loss of activity and that angular furanocoumarins are less active than linear furanocoumarins. The authors furthermore stated that the reported coumarins are not acting via the benzodiazepine binding site. Data of additional studies on coumarins confirms the reduced acitivity of angular furanocoumarins. In terms of receptor affinity instead, prenyl moieties show higher affinities than epoxyprenyl residues. As only one isocoumarin was reported to possess (moderate) GABAergic acitivity, no general conclusions can be drawn for this compound (sub)class.

#### 3.3.6. Diarylheptanoids, Stilbenes and Phenanthrenes

**Figure 9:**
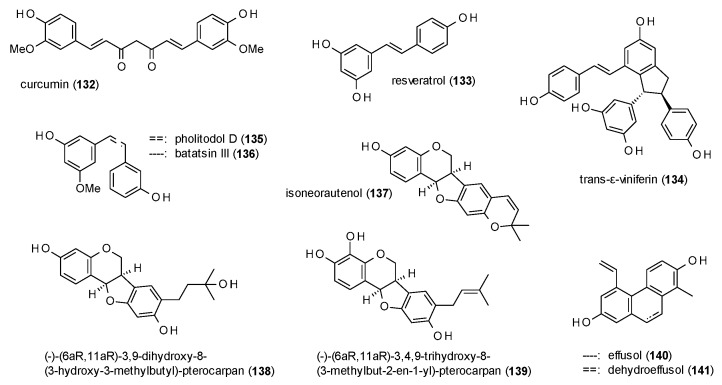
Chemical structures of diarylheptanoids, stilbenes and phenanthrenes with reported GABA(A)-receptor modulating activity.

This subsection contains the diarylheptanoid curcumin, two phenanthrenes, stilbenes and dihydrostilbenes (Figure 9).

Curcumin (**132**) was isolated from the oil of Curcumae rhizoma and tested on GABA(A) receptors expressed in HEK-293T cells, where the compound exhibited a maximal stimulation of 120% at a concentration of 50 µM [107].

Resveratrol (**133**), a prominent stilbene known from red wine, was studied by Hamid et al., together with trans-ε-viniferin (**134**) [108]. While resveratrol was found to moderately stimulate GABA-induced chloride currents (126% at a concentration of 100 µM), with an EC_50_ value of 58.24 µM, trans-ε-viniferin was identified as GABA(A) receptor inhibitor, with an IC_50_ value of 5.79 µM.

Rueda et al. isolated the stilbene pholitodol D (**135**) and its dihydro-derivative batatasin III (**136**) from *Pholidota chinensis* [109]. At a concentration of 300 µM both compounds showed a pronounced stimulation of GABA-induced chloride currents in the *Xenopus laevis* oocyte assay, with a maximal stimulation of 786.8% found for pholitodol D and 1512.9% for batatasin III, with EC_50_ values of 175.5 and 52.5 µM, respectively. The authors conclude that the conformational flexibility is the reason for the increased receptor stimulation of batatasin III, a theory which was confirmed by measuring 13 commercially available stilbenes and their corresponding dihydro-derivatives.

Another series of stilbenoids (**137**–**139**) with noteworthy activities was isolated from the roots and tubers from *Adenocarpus cincinnatus* [82]. At a concentration of 500 µM the compounds potentiated GABA-induced ion currents in a range from 491 to 771%, with EC_50_ values of 40.7 (**137**), 8.6 (**138**) and 18.8 µM (**139**).

Effusol (**140**) and dehydroeffusol (**141**) were isolated from *Juncus effusus* and found to modulate GABA(A) receptors with EC_50_ values of 31 and 27 µM [110]. At a concentration of 300 µM, the compounds exhibited maximal stimulations of 188 and 239%, respectively.

The increased receptor modulation of dihydrostilbenes in comparison to stilbenes has already been proven by studies with semi-synthetic dihydro-derivatives and is also evident in the two-fold higher potentiation and the three times lower EC_50_ of batatasin III (**136**) compared to pholitodol (**135**). Prenylated dihydrostilbenes (**137**–**139**) show a maximal potentiation in the range of pholitodol, but lower EC_50_ values than both before mentioned compounds. Here, the compound bearing an open oxyprenylated side chain (**138**) shows the lowest value, followed by prenylated compound **139** and isoneorautenol (**137**), which has a cyclized prenyl moiety.

#### 3.3.7. Simple Phenols and Polyphenols

**Figure 10:**
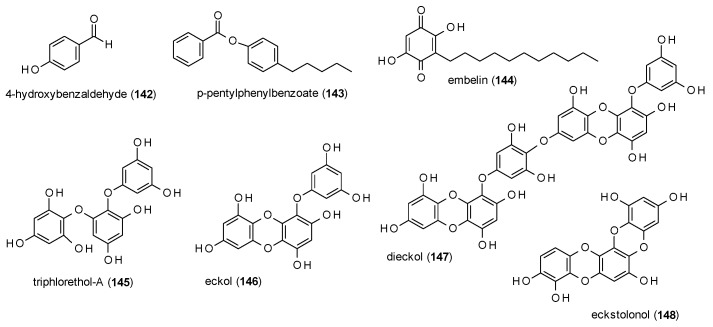
Chemical structures of simple phenols and polyphenols with reported GABA(A)-receptor modulating activity.

In this subsection, simple phenols and their condensed forms will be discussed (Figure 10).

4-Hydroxybenzaldehyde (**142**) was identified as one of two active principles in an aqueous extract of *Gastrodia elata*, by increasing the number of open arm entries at doses 50 and 100 mg/kg and time spent in the open arm at a dose of 100 mg/kg in the EPM test [111]. In contrast to the other active constituent, 4-hydroxybenzylalcohol (which was antagonized by WAY-100635), the effects of **142** were blocked by flumazenil.

p-pentylphenylbenzoate (**143**) was isolated from the Nigerian medicinal plant *Mondia whitei* and was found to decrease exploratory activity in the holeboard test and locomotor activity, rearing and grooming in the open field test at doses of 100, 200 and 300 mg/kg [112]. The compound also increased the time spent in the open arms of an EPM test at a dose of 300 mg/kg. However, GABA(A) receptor binding was only suggested by a computational model and not confirmed by co-application of a GABA(A) receptor antagonist.

Embellin (**144**), a benzoquinone isolated from *Embelia ribes*, was reported to have anticonvulsant activities [113]. In the MES-induced seizure model, embelin caused a reduction of hindlimb tonic extensions at doses of 2.5 and 5 mg/kg. At a dose of 10 mg/kg no hindlimb tonic extensions were observed. In the PTZ-induced seizures model embelin increased the onset of clonic and tonic action at doses of 2.5, 5 and 10 mg/kg and exhibited 50% (5 mg/kg) and 83% (10 mg/kg) protection against mortality. Embellin also reduced locomotor activity at all three doses.

Cho et al. isolated four phlorotannis (**145**–**148**) from the edible brown seaweed *Ecklonia cava* and measured their GABA(A) receptor affinity against [^35^S]TBPS [114]. The obtained Ki values were 4.419 (triphlorethol-A), 1.070 (eckol), 3.072 (dieckol) and 1.491 µM (eckstolonol), and IC_50_ values were calculated with 7.180, 1.739, 4.991 and 2.422 µM, respectively.

The reported polyphenolic compounds show comparable receptor affinities. Because of this and the fact that the same structural features appear repeatedly, a discussion on the structure-acitivity relationship of this compound class seems pointless.

### 3.4. Terpenes

#### 3.4.1. Monoterpenes

**Figure 11:**
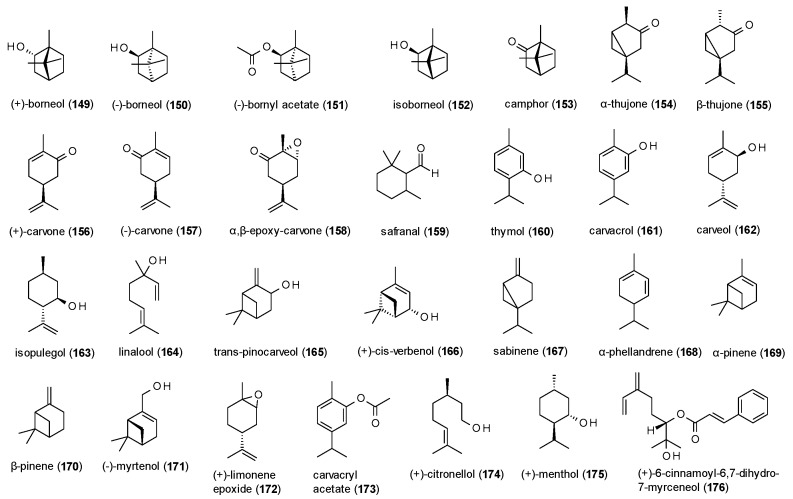
Chemical structures of monoterpenes with reported GABA(A)-receptor modulating activity.

A total of 28 monoterpenes with GABA(A) receptor modulating activity have been reported, including one cinnamoyl derivative (Figure 11).

Granger et al. investigated (+)-borneol (**149**), (−)-borneol (**150**), (−)-bornyl acetate (**151**), isoborneol (**152**) and camphor (**153**) on GABA(A) receptors expressed in *Xenopus laevis* oocytes [115]. All compounds caused pronounced maximal potentiation of GABA-induced chloride currents, with the highest stimulation observed for (+)-borneol (1251%) and (−)-borneol (1106%). However, EC_50_ values were in a high micromolar range with the lowest value observed for bornyl acetate (111.2 µM). (−)-borneol was also studied in vivo, where it showed a prolongation of sleeping time and reduced sleeping latency in the phenobarbital-induced sleeping test at doses from 5 to 20 mg/kg p.o. [116]. At doses of 50, 100 and 200 mg/kg p.o. (−)-borneol increased latency and protection against PTZ-induced convulsions, an effect that was antagonized by flumazenil.

Höld et al. tested α-thujone (**154**) and β-thujone (**155**) against [^3^H]EBOB in a radioligand binding assay, where the compounds showed IC_50_ values of 13 and 37 µM [117]. Additional electrophysiological investigations identified α-thujone as non-competitive antagonist with an IC_50_ value of 21 µM. The allosteric reduction of GABA-induced chloride currents was confirmed by Czyzewska et Mozrzymas, who measured a decrease in stimulation of 60% at a concentration of 300 µM [118]. In a study on GABAergic miniature inhibitory currents, α-thujone was found to reduce their frequency and amplitude and moderately affected their kinetics. Because the compound also reduced the amplitude of current responses to exogenous GABA and affected their onset, desensitization and deactivation, the authors assume α-thujone to have receptor gating effects [119]. A mixture of both enantiomers was tested for their effect on GABA-induced enhancement of [^3^H]flunitrazepam binding [120]. Thujone reduced the maximal enhancement of GABA from 182 to 103%, confirming the negative allosteric mechanism of the compound. In the same study, (+)-carvone (**156**) and (−)-carvone (**157**) were investigated. While (−)-carvone decreased enhancement of GABA to only 116%, (+)-carvone was even more effective than thujone, with a decrease to 74%.

α,β-epoxy-carvone (**158**) was investigated for its anticonvulsant properties using MES-, PTZ- and picrotoxin-induced seizure models [121]. α,β-epoxy-carvone was protective against MES- and picrotoxin-induced seizures at doses of 200, 300 and 400 mg/kg and increased the latency of picrotoxin-induced seizures at 300 and 400 mg/kg. At the latter doses, the compound was also protective against PTZ-induced seizures. The observed anticonvulsant effects were not affected after pretreatment with flumazenil.

Safranal (**159**) also showed anticonvulsant effects in the PTZ-induced seizures model, where it decreased the incidence of minimal clonic seizures and generalized tonic clonic seizures at a dose of 145.5 mg/kg and increased latency of both types [122]. Flumazenil abolished the protective effects of safranal, indicating involvement of GABA(A) receptors. However, after intracerebroventricular injection of safranal, no anticonvulsant effects were observed.

Thymol (**160**), an aromatic monoterpene known from several *Thymus* species, was investigated on α_1_β_3_γ_2_ receptors expressed in *Xenopus* oocytes, where it potentiated GABA-induced chloride currents by 416% at a concentration of 100 µM [123]. It also evoked small currents in the absence of GABA. Garcia et al. investigated the effects of thymol on [^3^H]flunitrazepam binding [124]. Thymol thereby caused a maximum response of [^3^H]flunitrazepam binding, with an EC_50_ value of 130.9 µM.

Kessler et al. tested twelve monoterpenes (**161**–**171**) contained in an extract of *Sideritis* sp. on GABA(A) receptors expressed in both, *Xenopus laevis* oocytes and HEK-293T cells [33]. Of the tested compounds (+)-cis-verbenol (**166**) and (−)-myrtenol (**171**) showed pronounced effects, potentiating GABA-induced chloride currents by 809% and 737%, respectively.

Isopulegol (**163**) was investigated in vivo for its anxiolytic potential and was found to increase the number of head dips in the holeboard test at doses of 25 and 50 mg/kg [125]. At the same doses isopulegol increased the number of open arm entries and time spent in the open arms and decreased the number of closed arm entries and time spent in closed arms in the EPM test. However, at theses doses an increased immobility time in the forced swim test and the tail suspension test were also observed.

Viana et al. studied (−)-myrtenol (**171**) for its gastroprotective effect against ethanol-induced acute gastric lesions [126]. The compound caused a decrease of gastric lesions at doses of 25, 50 and 100 mg/kg p.o. Pretreatment with flumazenil (20 mg/kg) reduced the gastroprotective effect of 50 mg/kg (−)-myrtenol.

In a study on the anxiolytic-like action of (+)-limonene expoxide (**172**), the compound showed an increase in open arm entries and time spent in the open arms at doses of 25, 50 and 75 mg/kg in the EPM test [127]. At the same doses a reduced number of crossing, rearing and grooming was observed in the open field test, furthermore indicating sedative effects of the compound. At a dose of 75 mg/kg increased number of falls and reduced time of permanence on the bar were measured, an effect that was reversed by flumazenil. The anxiolytic-like effect of (+)-limonene expoxide was confirmed by a follow-up study, where the compound showed a reduction in the number of buried marbles at 25, 50 and 75 mg/kg in the buried marbles test [128].

Also, carvacryl acetate (**173**) was investigated for its anxiolytic-like and sedative effects in several experiments [129]. In the EPM test the compound increased the number of open arm entries at a dose of 100 mg/kg and the time spent in the open arm at doses from 25 to 100 mg/kg. In the light/dark test carvacryl acetate increased the number and time spent in the light area at doses from 25 to 100 mg/kg. Both effects were blocked by flumazenil (25 mg/kg) but not by WAY-100635 (10 mg/kg). In the buried marbles test, a reduced number of buried marbles was observed at doses from 25 to 100 mg/kg, but no coordination impairment in the rotarod test and no decrease in locomotor activity in the open field test was measured at the same doses.

(+)-citronellol (**174**) was studied for its anticonvulsant effects using the MES-, PTZ- and picrotoxin-induced seizure models [130]. (+)-citronellol induced the latency of PTZ-induced seizures at doses of 100, 200 and 400 mg/kg and reduced the number of convulsions at a dose of 400 mg/kg. At this dose the compound was also protective against tonic extensions in the MES-induced seizures model. In the picrotoxin-induced seizures test, (+)-citronellol showed an increased latency at doses of 200 and 400 mg/kg.

Electrophysiological studies on (+)-menthol (**175**), using the *Xenopus laevis* oocyte assay, revealed a potentiation of GABA-induced chloride currents of 496% at a concentration of 100 µM [131] and 96.2% at a concentration of 50 µM, respectively [132]. The latter study determined an EC50 value of 23.5 µM and found the binding site of (+)-menthol to be distinct from benzodiazepines, steroids and barbiturates, but similar to that of propofol.

The last monoterpene to be discussed is (+)-6-cinnamoyl-6,7-dihydro-7-myrceneol (**176**), which was isolated from *Kadsura longipedunculata* and showed a maximum stimulation of GABA-induced chloride currents of 834.6%, with an EC_50_ value of 70.6 µM [96].

Several monoterpenes have been investigated for their GABA receptor modulating activity and thereof the highest potentiation of chloride currents was observed for bicyclic alocohols, like (+)- and (−)-borneol (**149**,**150**), with maximal stimulations of more than 1100%. However, isoborneol (**152**), (+)-cis-verbenol (**166**) and (−)-myrtenol (**171**) also exhibited pronounced potentiation. Oxidation of the hydroxy-group or the presence of an exocyclic methylene group decreased the activity, as well as ring opening. The only monocyclic monoterpenes with noteworthy positive receptor modulation are thymol and carveol (**160**,**162**), eliciting a potentiation of over 400%. Interestingly, when oxidizing the latter compound into carvone (**156**,**157**), the positive allosteric modulation is inverted, and chloride currents are allosterically inhibited instead. An effect also observed both, α- and β-thujone (**154**,**155**).

#### 3.4.2. Sesquiterpenes

**Figure 12:**
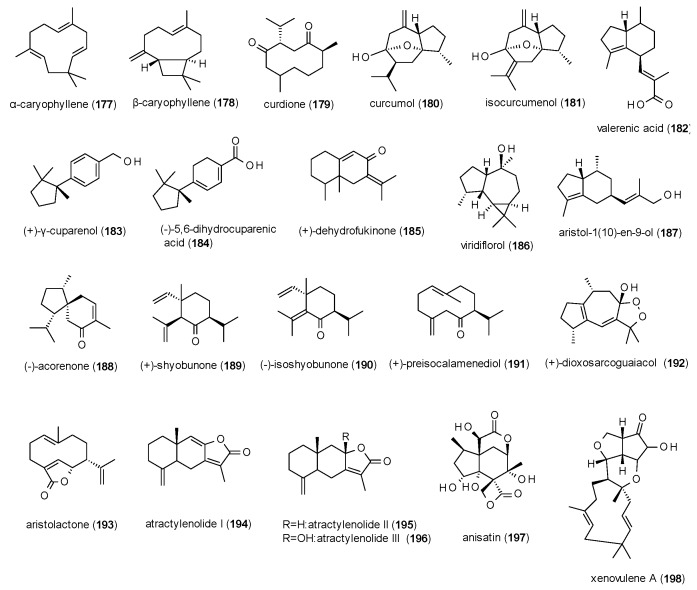
Chemical structures of sesquiterpenes with reported GABA(A)-receptor modulating activity.

A total of 28 sesquiterpenes with GABA(A) receptor modulating activity have been reported, including one cinnamoyl derivative (Figure 12).

α-caryophyllene (**177**) and β-caryophyllene (**178**) were part of the study on monoterpenoids from *Sideritis* sp. by Kessler et al., where they showed only moderate potentiation of GABA-induced chloride currents (117 and 115%, respectively) [33].

Curdione (**179**) and curcumol (**180**) were isolated from the oil of Curcumae rhizoma and measured on GABA(A) receptors expressed in HEK-293T cells [107]. At a concentration of 50 µM the compounds potentiated GABA-induced chloride currents with 133 and 175.7%, respectively. Curcumol was further investigated, showing an EC_50_ value of 34.4 µM and a maximal stimulation of 251% (at a concentration of 300 µM). The compound also reduced locomotor activity in the open field test at a dose of 100 mg/kg. In a recent study by Liu et al., curcumol was reported to allosterically modulate the receptor at a binding site distinct from benzodiazepines [133].

Isocurcumenol (**181**), isolated from *Cyperus rotundus*, was analysed for its affinity to GABA(A) receptors using [^3^H]flumazenil and [^3^H]flunitrazepam, where it inhibited binding of the two ligands by 58.3 and 105.0%, respectively (at a concentration of 100 µM) [134].

Valerenic acid (**182**), isolated from *Valeriana officinalis*, caused a potentiation of GABA-induced chloride currents of 400% (at a concentration of 100 µM) with an EC_50_ value of 13.6 µM [135], thus confirming earlier reports of the possible GABAergic mechanism of valerian extracts [136]. In an EPM test, valerenic acid prolonged the time spent in the open arms at a dose of 3 mg/kg [137].

(+)-γ-cuparenol (**183**) and (+)-dihydrocuparenic acid (**184**), isolated from *Kadsura longipedunculata*, exhibited a maximal stimulation of GABA-induced chloride current of around 400%, with EC_50_ values of 57.3 and 118.4 µM, respectively [96].

(+)-dehydrofukinone (**185**), isolated from *Nectandra grandiflora*, was measured in the PTZ-induced seizures model, where it delayed the onset of myoclonic jerks and generalized tonic clonic seizures at a dose of 10 mg/kg p.o. and reduced the latency to generalized seizures at 10 and 100 mg/kg p.o. [138].

Viridoflorol (**186**) was isolated from *Mentha aquatica* and subjected to a radioligand binding assay using [^3^H]flumazenil, but showed only weak receptor affinity with an IC_50_ value of 190 mM [78].

Aristol-1(10)-en-9-ol (**187**) from *Nardostachys chinensis*, now classified as *Nardostachys jatamansi*, caused a prolongation of sleeping time in the pentobarbital-induced sleeping test after inhalation of a dose of 300 µg/cage [139]. The effect was antagonized by flumazenil (3 mg/kg).

Five sesquiterpenes (**188–192**), isolated from the rhizomes of *Acorus calamus*, were measured in the *Xenopus* oocyte assay, where they potentiated GABA-induced chloride currents from 164 to 886% at concentrations of 300 to 1000 µM, with the lowest IC_50_ value calculated for (−)-acorenone (**188**, 34.0 µM [87].

Aristolactone (**193**), a compound known from *Aristolochia manshuriensis*, was isolated from an adulterated commercial sample of *Bupleurum chinense* [140]. The compound was found to only moderately potentiate GABA-induced chloride currents with a maximal stimulation of 70.7% at a concentration of 1000 µM. The IC_50_ value of the compound was calculated as 56.02 µM.

Atractylenoids I (**194**), II (**195**) and III (**196**) were isolated from *Atractylodes macrocephala* and tested on GABA(A) receptors expressed in *Xenopus laevis* oocytes [141]. At concentrations of 300 µM the three compounds showed maximal potentiation of 96 to 166%, with EC_50_ values of 12, 70 and 99 µM, respectively.

Anisatin (**197**), a highly oxygenated sesquiterpene lactone from *Illicium anisatum*, was already described as potent non-competitive antagonist of GABA(A) receptor in 1981, with similar effects to those of picrotoxin [142,143]. Later studies in dorsal root ganglia found that anisatin at a concentration of 1 µM reduced chloride currents evoked by 30 µM GABA by 41.7% [144]. The IC_50_ value was calculated with 1.10 µM. In the same study, an IC_50_ value of 0.42 µM was determined for picrotoxinin, the (more) active compound of picrotoxin. In a radioligand binding assay anisatin showed an IC_50_ value of 0.43 µM against [^3^H]EBOB, indicating that anisatin binds to the picrotoxin site of the receptor.

Another very potent sesquiterpene is xenovulene A, which has been isolated from the fungus *Acremonium strictum* (now classified as *Sarocladium strictum*) [145]. The compound potentiated GABA-induced chloride currents by 180% at a concentration of 1 µM and showed an EC_50_ value at a nanomolar level (0.05 µM).

Due to the structural differences of the reported sesquiterpenes only limited conclusions on their structure-activity relationship can be drawn. One finding is that reduction of the acidic function of compound **184** to an alcoholic function (**183**) does not seem to alter the acitivity, while the change of the isopropenyl-function of compound **189** to a plane isopropanyl-moiety (**190**) leads to a significant loss of activity.

#### 3.4.3. Diterpenes

**Figure 13:**
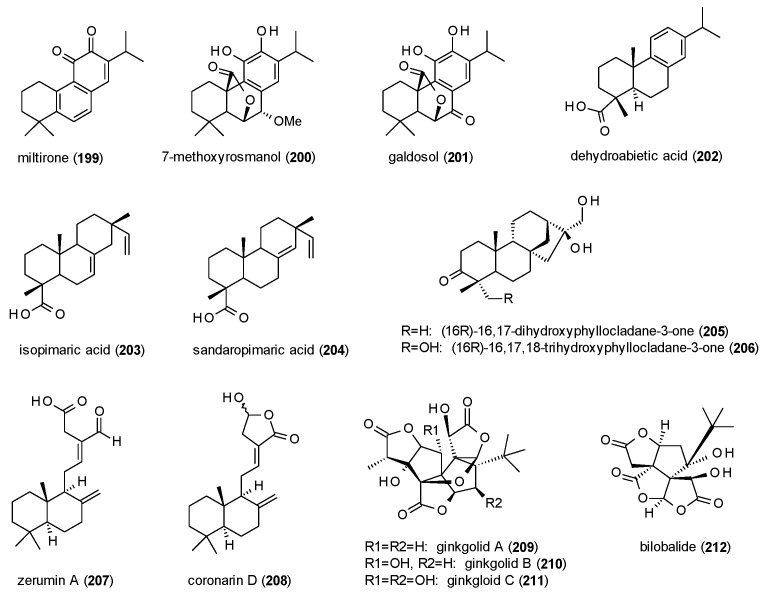
Chemical structures of diterpenes with reported GABA(A)-receptor modulating activity.

A total of 14 diterpenes with GABAergic activity will be discussed in this subsection (Figure 13).

Miltirone (**199**), a tanshinone from *Salvia miltiorrhiza*, was tested against [^3^H]flunitrazepam in a radioligand binding assay, exhibiting an IC_50_ value of 0.3 µM [146]. In subsequent in vivo studies, the compound increased the number of punished crossings in the four plates test at doses from 10 to 60 mg/kg p.o., but at the same doses showed no muscle relaxant effect and no impairment in the horizontal wire test. Due to these findings the authors concluded miltirone to be acting as a partial agonist on the benzodiazepine bindings site. These conclusions were confirmed by Mostallino et al., who additionally reported that miltirone does not potentiate GABA-induced ion currents in *Xenopus laevis* oocytes, but concentration-dependently inhibits the potentiation caused by diazepam [147].

7-methoxyrosmanol (**200**) and galdosol (**201**), two compounds isolated from *Salvia officinalis*, were analysed in a radioligand assay using [^3^H]flumazenil [63]. IC_50_ values were calculated as 7.2 and 0.8 µM, respectively.

Dehydroabietic acid (**202**) was isolated from *Boswellia thurifera*, now classified as *Boswellia serrata*, and analysed in *Xenopus laevis* oocytes [148]. The compound potentiated GABA-induced chloride currents by 397.5% at 100 µM and showed an EC_50_ value of 8.7 µM.

Isopimaric acid (**203**) and sandaropimaric acid (**204**) were isolated from *Biota orientalis*, which is meanwhile classified as *Platycladus orientalis*, and analysed in the *Xenopus* oocyte assay [149]. The compounds, at a concentration of 500 µM, exhibited a maximal potentiation of GABA-induced chloride currents of 425.2 and 855.7% and showed EC_50_ values of 141.6 and 33.2 µM, respectively. Maximal stimulation of chloride currents by both compounds was raised to over 1000%, when α_1_-subunits were exchanged for α_2_- or α_3_-subunits. 

Two phyllocladane-type diterpenes (**205**,**206**) were isolated from *Aloysia virgata* and tested for their GABA(A) receptor affinity against [^3^H]flumazenil, showing Ki values of 111 and 56 µM, respectively [150]. Both compounds were subsequently analysed in vivo, with compound **205** exhibiting increased locomotor activity at a dose of 1 mg/kg in the locomotor activity test and increased rearing at 0.3 and 1 mg/kg in the holeboard test. Compound **206** increased the number of head dips at 0.3 and 3 mg/kg, the number of rears at a dose of 1 mg/kg and the time spent head-dipping at a dose of 3 mg/kg. Moreover, the compound at a dose of 1 mg/kg increased the number of open arm entries in the EPM test and the time spent in the light area as well as the number of transitions in the light/dark test.

Zerumin A (**207**) and coronarin D (**208**), two labdan-type diterpenes, were isolated from *Curcuma kwangsiensis* [151]. The compounds, at a concentration of 300 µM, potentiated GABA-induced chloride currents in the *Xenopus* oocyte assay by 309.4 and 211.0%, respectively, with EC_50_ values of 24.9 and 35.7 µM.

Ginkgolides A (**209**), B (**210**) and C (**211**), diterpene trilactones from *Ginkgo biloba*, were found to be moderately potent antagonists at GABA(A) receptors expressed in *Xenopus laevis* oocytes, with Ki values of 14.5, 12.7 and 16.3 µM, respectively [152]. IC_50_ values of the three compounds were 11.9, 10.1 and 12.0 µM, respectively. A study on ginkgolide B and bilobalide (**212**) using cortical neurons found the compounds to inhibit chloride currents by 63.2 and 46.8% at a concentration of 50 µM [153]. IC_50_ values of the compounds were 73 and 76 µM, respectively. A radioligand binding assay of bilobalide using [^35^S]TBPS determined a Kd value of 3.7 µM [154]. In the same study the impact on the flux of ^36^chloride was measured. Bilobalide, at a concentration of 1000 µM totally blocked chloride uptake into synaptoneurosomes but showed only low potency with an IC_50_ value of 39 µM.

As for the sesquiterpenes, only a few findings can be gathered for the diterpenes from the reported data. One observation relates to the receptor affinity of compounds **200** and **201**, which is increased almost 10-fold by an oxo-group instead of an methoxy-group in position 7. For compounds **203** and **204**, one the other hand, the shift of a double bond from position 7 to 8, and thus into ring C of the compound, doubles the maximal potentiation and markedly reduces the EC_50_ value. Regarding the inhibitory acitivity of bilobalide and ginkgolids A-C, no clear differences can be observed in their IC_50_ values, nor in their ability to block GABA-induced chloride currents.

#### 3.4.4. Triterpenes

**Figure 14:**
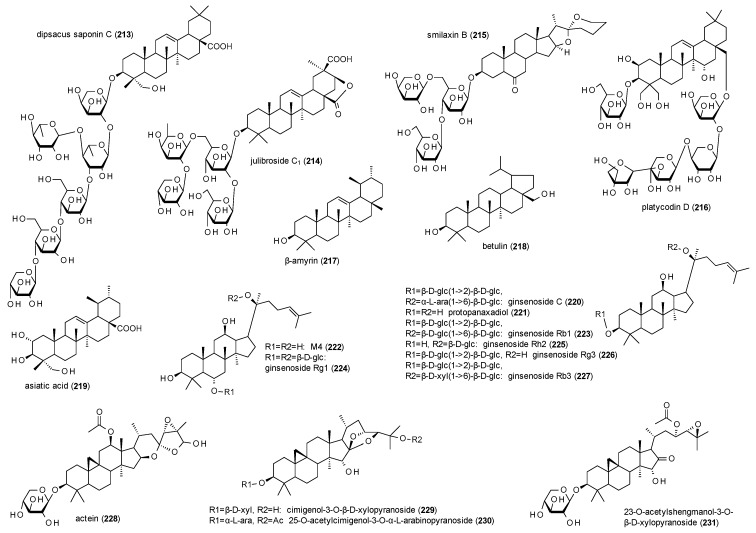
Chemical structures of triterpenes with reported GABA(A)-receptor modulating activity.

The last compound class to be discussed in this review are triterpenes and, most of all, triterpene glycosides (Figure 14).

Dipsacus saponin C (**213**), an oleanol-type triterpenoid isolated from *Dipsacus asper*, showed inhibition of tail-flick responses after intrathecal administration of 3.75 to 30 µg of the compound [155]. Pretreatment with 5-aminovaleric (1 to 20 µg i.c.v.) and gabazine (0.1 to 2 ng i.c.v.) both attenuated the inhibition induced by compound **213.**

Julibroside C_1_ (**214**) was isolated from *Albizia julibrissin* and investigated for its anxiolytic potential [156]. In the EPM test, the compound increased the number of open arm entries and time spent in open arms at doses of 0.5 and 1 mg/kg p.o., but had no effect on distance travelled. Flumazenil (10 mg/kg) and bicuculline (3 mg/kg) reduced the time spent in open arms.

Smilaxin B (**215**), was isolated from *Smilax sieboldii* and tested in the tail flick response assay, where it showed an inhibition of tail flick responses at doses from 7.5 to 30 µg i.c.v. [157]. The inhibition reached a peak at 7.5 min and lasted for 45 min. Muscimol (50 ng i.c.v.) reduced the inhibition induced by 30 µg smilaxin B.

Platycodin D (**216**), from *Platycodon grandiflorus*, also showed antinociceptive properties in the tail flick response test [158]. Inhibition of tail flick responses were observed at doses from 0.5 to 2 µg i.c.v., reached a peak at 15 min and lasted for at least 1 h. Muscimol at doses from 50 to 200 ng i.c.v. antagonized the effect.

β-amyrin (**217**) was investigated in the pentobarbital induced sleep model by Jeon et al. [159]. The compound exhibited a prolongation of sleep duration at a dose of 10 mg/kg p.o. and decreased the onset time at doses of 3 and 10 mg/kg p.o. Both effects were reversed by bicuculline (0.3 mg/kg i.p.). β-amyrin was additionally showed a decrease of locomotor activity in the open field at a dose of 10 mg/kg p.o.

Betulin (**218**) was analysed in a radioligand binding assay using [^3^H]GABA and showed a Ki value of 64 µM [160]. The compound was subsequently analysed for its protection against bicucullin-induced seizures and found to inhibit the effect of bicuculline at doses of 50 and 100 mg/kg. The protective effect of betulin was also observed after i.c. injection of 10, 50 or 100 nmole/mice.

Asiatic acid (**219**) was isolated from *Centella asiatica* and tested for its anxiolytic potential in the EPM [161]. The compound had no effect on the percentage of time spent in the open arms but decreased the time mobile and the maximum speed at doses of 30 mg/kg. Co-application of flumazenil antagonised both effects.

Ginsenoside C (**220**), one of several glycosides from *Panax ginseng*, was tested on GABA(A) receptors expressed in *Xenopus laevis* oocytes and found to potentiate GABA-induced chloride currents with an EC_50_ value of 53.2 µM [162].

Lee et al. reported lower EC_50_ values for the two aglycons protopanaxadiol (**221**) and M4 (**222**), which were calculated as 23.1 and 17.1 µM respectively [163]. However, both compounds only exhibited a weak potentiation of GABA-induced chloride currents.

Ginsenoside Rb1 (**223**) and Rg1 (**224**) were investigated for their anxiolytic-like effects in the EPM test [164]. Both compounds increased the number of open arm entries at doses of 10, 25 and 50 mg/kg p.o. and time spent in the open arms at 50 mg/kg p.o. Ginsenoside Rb1 additionally increased the time spent in the open arms at a dose of 25 mg/kg and showed a decrease of locomotor activity at a dose of 50 mg/kg p.o. in the locomotor activity test.

Ginsenoside Rh2 (**225**) and Rg3 (**226**) were also analysed in the EPM test showing an increased number of open arm entries at doses of 10 and 25 mg/kg p.o. and time spent in the open arms at a dose of 25 mg/kg p.o. [165]. Ginsenoside Rh2 additionally increased the time spent in the open arms at a dose of 10 mg/kg p.o.

Ginsenoside Rb3 (**227**) was investigated for its neuroprotective effect and found the compound to be acting in a similar way as muscimol [166]. Because both, bicucullin and picrotoxin prevented neuroprotection, the authors concluded the neuroprotective effects to be mediated via the GABA(A) receptor.

Four cycloartane glycosides (**228**–**231**) were isolated from the rhizomes of *Actaea racemosa* and measured for their potentiation of GABA-induced chloride currents in *Xenopus laevis* oocytes [167]. At a concentration of 300 µM, compounds **228**–**230** showed a potentiation of GABA-induced chloride currents in a range from 256 to 378%, while 23-*O*-acetylshengmanol-3-*O*-β-d-xylopyranoside (**231**) exhibited a stimulation of 1947% and was also found to produce small chloride currents in the absence of GABA. EC_50_ values of the four glycosides were calculated from 26 to 36 µM. Interestingly, cleavage of the pentose moiety led to a significant decrease of activity (especially for compound **231**). Compound **231** was subsequently subjected to several in vivo studies to examine its anxiolytic and sedative properties [168]. Compound **231** increased the number of open entries at 0.6 mg/kg in the EPM test and reduced stress-induced hyperthermia at doses of 0.2, 0.6, 2 and 6 mg/kg. In the open field test the compound reduced the distance travelled at doses of 6, 20 and 60 mg/kg and increased the time spent in the centre at a dose of 60 mg/kg, while the number of entries into the centre was reduced. Moreover, the compound was found anticonvulsant in the PTZ-induced seizures model, elevating the seizure threshold at doses of 20 and 60 mg/kg.

In contrast to the last two subsections, the structure-acitivity discussion of the triterpenes is not hindered by the lack of comparable structures (scaffolds), but by the number of different test systems used for their examination. However, at least some of the reported triterpenes from ginseng (**220**–**222**) and black coshosh (**228**–**231**) can be compared within and to each other. Electrophysiological recordings of the three ginseng tritperpenes revealed lower EC_50_ concentrations for the two aglycons (**221**,**222**) compared to ginsenoside C (**220**). Alas, values for the maximal potentiation of chloride currents were only found for the two aglycons and reported to be 54.1 and 23.3%, respectively (at a concentration of 100 µM). That the receptor modulation of the glycoside would be of great interest can be concluded after looking at compound **231**, where cleavage of the xylose moiety shifted the potentiation of GABA-induced chloride currents from 1692% to 64% (at 100 µM) and thus into the range of the ginseng aglycons. When comparing their scaffolds, compound **231** as well as the ginsenosides show a four-ring system with a side chain attached to ring D. This side chain reminds of the structure-activity-relationship of coumarins, where prenylated and oxyprenylated side chains increased the activity. In the case of the discussed triterpenes, the ginsenoside side-chain would stand for the prenyl moiety and that of compound **231** for the more potent epoxylated form. However, compound **231** shows additional features that could contribute to its pronounced effect, like the keto-function in position 16 or the acetyloxy-group in at C-23, which both distinguish the compound from the other, markedly less active, cycloartanoids (**228**–**230)**. Regarding the possible binding site, the neurosteroid binding site would be most obvious and in line with the fact that neurosteroids are the most potent natural GABA(A) receptor modulators and are furthermore able to evoke chloride currents in the absence of GABA [19,169]. However, for the neurosteroid binding site a hydroxy-group in position 3 and a keto-group in position 17 or 20 are considered essential for the activity [170]. Regarding the structure of compound **231**, the keto-group in position 16 instead of position 17 may well contribute to receptor binding but the fact that the compound’s activity almost disappears with the xylose moiety, does not support this theory, unless a sugar residue instead of a hydroxy-group in position 3 would be able to increase binding to the neurosteroid site. On the other hand, also barbiturates are known to directly activate GABA(A) receptors at higher concentrations and the barbiturate binding site is hypothesized to be similar to that for neurosteroids [19].

## 4. Conclusions

A total of 231 natural products with activity on GABA(A) receptors were found in the literature and discussed in this review. Depending on the number of similar compounds and test systems used, more or fewer conclusions on their structure-activity relationships were possible. As most studies investigated flavones, and these studies mainly applied radioligand binding assays, the substitution patterns responsible for increased receptor affinity could be identified, with one flavone even showing diazepam-like Ki values. In terms of receptor modulation, flavones were found to be either non-competitive antagonists or partial agonists. However, some compounds still exhibited anxiolytic or anticonvulsive effects. Other phenolic compounds discussed in this review were e.g., coumarins, where prenylated compounds showed higher receptor stimulation. The correlation of prenyl residues and pronounced receptor modulation was also observed for flavanes, isoflavonoids and chalcones and might be interesting for the development of GABA(A) receptor modulators.

Moreover, the necessary structural features for the positive or negative receptor modulation of polyacetylenes and monoterpenes were highlighted, as well as the effect of deglycosylation in the case of some triterpenes. Regarding subtype-specifity of natural products, only limited reports have been found. One example is the enhanced receptor modulation of isopimaric and sandaropimaric acid after exchange of the α1-subunit for α2- or α3-subunits. In this context, the neolignane honokiol must also be mentioned, although the effect was more dependent on the β-subunits of the GABA(A) receptor. Here, the collected data of reported in vivo studies might be helpful, as several compounds have been identified to exhibit anxiolytic effects without showing sedative or muscle relaxant properties.

## Figures and Tables

**Table 1:** Data from electrophysiological studies of GABA(A) receptor antagonists.

Cmpd	Source	Assay	Subtype	c(GABA)	I_max_ [%]	c [µM]	IC_50_ [µM]	Ref.
**1**	*Colchicum autumnale*	*Xenopus* oocytes	α_1_β_2_γ_2L_	10 µM	59.9 ± 6.2	100		[23]
**19**	*Aconitum leucostomum*	Hippocampal neurons					19.6	[31]
**22**	*Oenanthe fistulosa*	HEK-293T cells	α_1_β_2_γ_2L_				1.39	[35]
**23**	*Oenanthe fistulosa*	HEK-293T cells	α_1_β_2_γ_2L_				0.835	[35]
**26**	*Cicuta virosa*	Hippocampal neurons		10 µM	90	10	0.96	[37]
**34**		*Xenopus* oocytes	α_1_β_2_γ_2S_		14.8 ± 0.8	30		[54]
**45**		*Xenopus* oocytes	α_1_β_2_γ_2S_		12.1 ± 0.5	30		[54]
**56**	*Scuttelaria baicalensis*	CA1 neurons		10 µM	38.2 ± 6	100		[46]
**63**		*Xenopus* oocytes	α_1_β_2_γ_2S_		50.6 ± 0.8	30		[54]
**63**	*Matricaria recutita*	Cerebellar granule cells		10 µM	32 ± 4	10		[55]
**63**		*Xenopus* oocytes	α_1_β_2_γ_2L_	40 µM	49.6	100	8	[56]
**63**		*Xenopus* oocytes	α_1_β_2_γ_2S_				6.9 ± 0.3	[57]
**64**		HEK-293T cells	α_1_β_2_γ_2_		66.5 ± 6.8		6.62 ± 2.11	[62]
**73**		*Xenopus* oocytes	α_1_β_2_γ_2S_		84.5 ± 4.9	30		[54]
**74**		*Xenopus* oocytes	α_1_β_2_γ_2S_		38.4 ± 4.8	30		[54]
**84**		*Xenopus* oocytes	α_1_β_2_γ_2L_	40/20 µM	90.6/97	100	14.7/8.7	[56]
**96**		*Xenopus* oocytes	α_1_β_2_γ_2L_	40/5 µM	51/40	100	29.2/11.7	[56]
**134**		*Xenopus* oocytes	α_1_β_2_γ_2L_				5.79	[108]
**154**		Dorsal root ganglia		300 µM	100	30	21	[117]
**154**		HEK-293T cells	α_1_β_2δ_		60	300		[118]
**197**	*Illicium anisatum*	Dorsal root ganglia		30 µM	41.7	1	1.10 ± 1.40	[144]
**209**	*Ginkgo biloba*	*Xenopus* oocytes	α_1_β_2_γ_2L_	300 µM			11.9 ± 1.7	[152]
**210**	*Ginkgo biloba*	*Xenopus* oocytes	α_1_β_2_γ_2L_	300 µM			10.1 ± 2.9	[152]
**210**	*Ginkgo biloba*	Cortical neurons	α_1_β_2_γ_2L_	30 µM	63.2 ± 0.3	50	73	[153]
**211**	*Ginkgo biloba*	*Xenopus* oocytes	α_1_β_2_γ_2L_	300 µM			12.0 ± 2.2	[152]
**212**	*Ginkgo biloba*	Cortical neurons	α_1_β_2_γ_2L_	30 µM	46.8 ± 0.3	50	76 µM	[153]

**Table 2:** Data from electrophysiological studies of GABA(A) receptor agonists.

Cmpd	Source	Assay	Subtype	c(GABA)	P_max_ [%]	c [µM]	EC_50_ [µM]	Ref.
**5**	*Piper nigrum*	*Xenopus* oocytes	α_1_β_2_γ_2S_	EC_5–10_	302 ± 26	300	52 ± 9	[25]
**6**	*Piper nigrum*	*Xenopus* oocytes	α_1_β_2_γ_2S_	EC_5–10_	187 ± 10	300	56 ± 19	[25]
**20**		*Xenopus* oocytes	α_1_β_2_γ_2_	1 µM	295 ± 50	300		[33]
**31–33**	*Cussonia zimmermannii*	*Xenopus* oocytes	α_1_β_2_γ_2_		110–440		0.6–3.5	[40]
**46**	*Scuttelaria baicalensis*	*Xenopus* oocytes	α_1_β_2_γ_2_	EC_1_	57 ± 6	30	3	[48]
**66**		Xenopus oocytes	α_1_β_2_γ_2_	EC_2–5_	47 ± 5	10		[57]
**82**		*Xenopus* oocytes	α_4_β_4_δ		68 ± 5	100		[76]
**88**	*Sophora flavescens*	*Xenopus* oocytes	α_1_β_2_γ_2S_	EC_3–10_	578.5 ± 68.8		8.1 ± 1.4	[79]
**89**	*Sophora flavescens*	*Xenopus* oocytes	α_1_β_2_γ_2S_	EC_3–10_	267.6 ± 56.6		5.0 ± 2.3	[79]
**90**	*Sophora flavescens*	*Xenopus* oocytes	α_1_β_2_γ_2S_	EC_3–10_	604.9 ± 108.2		15.0 ± 3.6	[79]
**92**	*Morus alba*	*Xenopus* oocytes	α_1_β_2_γ_2S_		730.4 ± 76.7	100	13.8 ± 1.5	[81]
**93**	*Morus alba*	*Xenopus* oocytes	α_1_β_2_γ_2S_		715.8 ± 56.1	100	16.7 ± 2.0	[81]
**94**	*Morus alba*	*Xenopus* oocytes	α_1_β_2_γ_2S_		719.3 ± 63.3	100	13.4 ± 1.6	[81]
**95**	*Adenocarpus cinncinatus*	*Xenopus* oocytes	α_1_β_2_γ_2S_	EC_5–10_	552.73 ± 84.07	500	2.8 ± 1.4	[82]
**97**	*Glycyrrhiza glabra*	Dorsal raphe neurons	α_2_β_2/3_γ_2L_	2 µM	581 ± 91	3		[83]
**98**		Dorsal raphe neurons		EC_10_	151	10		[84]
**99**	*Sophora flavescens*	*Xenopus* oocytes	α_1_β_2_γ_2S_	EC_3–10_	891.5 ± 163.0		4.0 ± 2.4	[79]
**101**		HEK-293T cells	α_1_β_2_γ_2_				290 ± 28	[86]
**102**	*Acorus calamus*	*Xenopus* oocytes	α_1_β_2_γ_2S_	EC_5–10_	1200 ± 163	500	171.5 ± 34.6	[87]
**103**	*Magnolia officinalis*	*Xenopus* oocytes	α_1_β_2_γ_2S_		1315 ± 281	300	36.2 ± 14.7	[88]
**106**		HEK-293T cells					10.1 ± 10.5	[93]
**107**		HEK-293T cells					16.9 ± 0.3	[93]
**108**		HEK-293T cells					10.5 ± 2.3	[93]
**109**		Cortical neurons		10 µM	158 ± 20	1	0.04258	[94]
**110**	*Piper methysticum*	Neonatal rat gastric-brainstem preparation			31.5 ± 3.9	300	93	[95]
**111**	*Kadsura longipedunculata*	*Xenopus* oocytes	α_1_β_2_γ_2S_	EC_5–10_	218.1 ± 20.8		21.8 ± 7.5	[96]
**112**	*Kadsura longipedunculata*	*Xenopus* oocytes	α_1_β_2_γ_2S_	EC_5–10_	245.0 ± 59.6		52.2 ± 24.8	[96]
**113**	*Kadsura longipedunculata*	*Xenopus* oocytes	α_1_β_2_γ_2S_	EC_5–10_	885.8 ± 291.2		135.6 ± 85.7	[96]
**114**	*Kadsura longipedunculata*	*Xenopus* oocytes	α_1_β_2_γ_2S_	EC_5–10_	168.7 ± 41.5		36.6 ± 16.4	[96]
**115**	*Kadsura longipedunculata*	*Xenopus* oocytes	α_1_β_2_γ_2S_	EC_5–10_	129.7 ± 36.8		118.7 ± 54.4	[96]
**116**	*Kadsura longipedunculata*	*Xenopus* oocytes	α_1_β_2_γ_2S_	EC_5–10_	395.6 ± 27.2		31.5 ± 7.1	[96]
**117**	*Kadsura longipedunculata*	*Xenopus* oocytes	α_1_β_2_γ_2S_	EC_5–10_	288.8 ± 23.7		12.8 ± 3.1	[96]
**118**	*Kadsura longipedunculata*	*Xenopus* oocytes	α_1_β_2_γ_2S_	EC_5–10_	793.4 ± 107.4		79.2 ± 19.4	[96]
**119**	*Kadsura longipedunculata*	*Xenopus* oocytes	α_1_β_2_γ_2S_	EC_5–10_	362.5 ± 87.1		54.6 ± 28.8	[96]
**121**	*Cnidium monnieri*	*Xenopus* oocytes	α_1_β_2_γ_2S_				54 ± 13	[99]
**121**	*Angelica pubescens*	*Xenopus* oocytes	α_1_β_2_γ_2S_	EC_5–10_	25.8 ± 12.7	300		[100]
**122**		*Xenopus* oocytes	α_1_β_2_γ_2S_				57 ± 4	[99]
**124**		*Xenopus* oocytes	α_1_β_2_γ_2S_		550 ± 71	100	25 ± 8	[99]
**125**		*Xenopus* oocytes	α_1_β_2_γ_2S_				34 ± 6	[99]
**126**	*Angelica pubescens*	*Xenopus* oocytes	α_1_β_2_γ_2S_	EC_5–10_	204.5 ± 33.2	300		[89]
**127**	*Cnidium monnieri*	*Xenopus* oocytes	α_1_β_2_γ_2S_		124 ± 11	100	14 ± 1	[99]
**127**	*Angelica pubescens*	*Xenopus* oocytes	α_1_β_2_γ_2S_	EC_5–10_	273.6 ± 39.4	300		[100]
**128**	*Angelica pubescens*	*Xenopus* oocytes	α_1_β_2_γ_2S_	EC_5–10_	61.2 ± 20.2	300		[100]
**129**	*Angelica pubescens*	*Xenopus* oocytes	α_1_β_2_γ_2S_	EC_5–10_	38.0 ± 21.3	300		[100]
**131**	*Haloxylon scoparium*	*Xenopus* oocytes	α_1_β_2_γ_2S_		144.6 ± 35.3	500	140.2 ± 51.2	[106]
**132**	*Rhizoma curcumae oil*	HEK-293T cells	α_1_β_2_γ_2_	1 µM	120 ± 6	50		[107]
**133**		*Xenopus* oocytes	α_1_β_2_γ_2L_	EC_10_ (3 µM)	126 ± 15	100	58.24	[108]
**135**	*Pholidota chinensis*	*Xenopus* oocytes	α_1_β_2_γ_2S_	EC_3–10_	786.8 ± 72.1	300	175.5 ± 25.5	[109]
**136**	*Pholidota chinensis*	*Xenopus* oocytes	α_1_β_2_γ_2S_	EC_3–10_	1512.9 ± 176.5	300	52.5 ± 17.0	[109]
**137**	*Adenocarpus cinncinatus*	*Xenopus* oocytes	α_1_β_2_γ_2S_	EC_5–10_	771.09 ± 57.94	500	40.7 ± 4.08	[82]
**138**	*Adenocarpus cinncinatus*	*Xenopus* oocytes	α_1_β_2_γ_2S_	EC_5–10_	640.02 ± 53.56	500	8.6 ± 1.6	[82]
**139**	*Adenocarpus cinncinatus*	*Xenopus* oocytes	α_1_β_2_γ_2S_	EC_5–10_	490.97 ± 22.34	500	18.8 ± 2.3	[82]
**140**	*Juncus effusus*	*Xenopus* oocytes	α_1_β_2_γ_2S_	EC_5–10_	188 ± 20	300	31 ± 8	[110]
**141**	*Juncus effusus*	*Xenopus* oocytes	α_1_β_2_γ_2S_	EC_5–10_	239 ± 18	300	27 ± 6	[110]
**149**		*Xenopus* oocytes	α_1_β_2_γ_2L_	EC_5–14_	1251 ± 73	300	247.7	[115]
**150**		*Xenopus* oocytes	α_1_β_2_γ_2L_	EC_15–24_	1106 ± 73	300	236.9	[115]
**151**		*Xenopus* oocytes	α_1_β_2_γ_2L_	EC_25–39_	571 ± 123	300	111.2	[115]
**152**		*Xenopus* oocytes	α_1_β_2_γ_2L_	EC_15–24_	968 ± 88	300	190.5	[115]
**153**		*Xenopus* oocytes	α_1_β_2_γ_2L_	EC_15–24_	377 ± 156	300	469.1	[115]
**160**		*Xenopus* oocytes	α_1_β_3_γ_2S_	EC_20_	416 ± 72	100		[123]
**161**		*Xenopus* oocytes	α_1_β_2_γ_2_	1 µM	224 ± 85	300		[33]
**162**		*Xenopus* oocytes	α_1_β_2_γ_2_	1 µM	453 ± 176	300		[33]
**163**		*Xenopus* oocytes	α_1_β_2_γ_2_	1 µM	340 ± 70	300		[33]
**164**		*Xenopus* oocytes	α_1_β_2_γ_2_	1 µM	213 ± 105	300		[33]
**165**		*Xenopus* oocytes	α_1_β_2_γ_2_	1 µM	477 ± 68	300		[33]
**166**		*Xenopus* oocytes	α_1_β_2_γ_2_	1 µM	809 ± 118	300		[33]
**167**		HEK-293T cells	α_1_β_2_γ_2_	1 µM	156 ± 26	1000		[33]
**168**		HEK-293T cells	α_1_β_2_γ_2_	1 µM	168 ± 42	1000		[33]
**169**		HEK-293T cells	α_1_β_2_γ_2_	1 µM	116 ± 56	1000		[33]
**170**		HEK-293T cells	α_1_β_2_γ_2_	1 µM	179 ± 55	1000		[33]
**171**		*Xenopus* oocytes	α_1_β_2_γ_2_	1 µM	737 ± 234	300		[33]
**175**		Xenopus oocytes	α_1_β_2_γ_2S_	EC_20_	496 ± 113	100		[131]
**175**		Xenopus oocytes	α_1_β_2_γ_2S_	EC_20_	96.2 ± 3.8	50		[132]
**176**	*Kadsura longipedunculata*	*Xenopus* oocytes	α_1_β_2_γ_2S_	EC_5–10_	834.6 ± 77.5		70.6 ± 12.2	[96]
**177**		HEK-293T cells	α_1_β_2_γ_2_	1 µM	117 ± 57	1000		[33]
**178**		HEK-293T cells	α_1_β_2_γ_2_	1 µM	115 ± 52	1000		[33]
**179**	Rhizoma curcumae oil	HEK-293T cells	α_1_β_2_γ_2_	1 µM	133 ± 10	50		[107]
**180**	Rhizoma curcumae oil	HEK-293T cells	α_1_β_2_γ_2_	1 µM	251 ± 16	300	34.4 ± 2.9	[107]
**182**	*Valeriana officinalis*	*Xenopus* oocytes	α_1_β_2_γ_2S_	EC_5–10_	400.0 ± 77.6	100	13.6 ± 4.1	[135]
**183**	*Kadsura longipedunculata*	*Xenopus* oocytes	α_1_β_2_γ_2S_	EC_5–10_	383.5 ± 89.3		57.3 ± 19.7	[96]
**184**	*Kadsura longipedunculata*	*Xenopus* oocytes	α_1_β_2_γ_2S_	EC_5–10_	413.4 ± 66.3		118.4 ± 29.9	[96]
**188**	*Acorus calamus*	*Xenopus* oocytes	α_1_β_2_γ_2S_	EC_5–10_	241 ± 23.1	500	34.0 ± 6.7	[87]
**189**	*Acorus calamus*	*Xenopus* oocytes	α_1_β_2_γ_2S_	EC_5–10_	669 ± 112	1000	64.8 ± 19.8	[87]
**190**	*Acorus calamus*	*Xenopus* oocytes	α_1_β_2_γ_2S_	EC_5–10_	164 ± 42.9	500	109.4 ± 46.6	[87]
**191**	*Acorus calamus*	*Xenopus* oocytes	α_1_β_2_γ_2S_	EC_5–10_	886 ± 105	1000	135.1 ± 34.4	[87]
**192**	*Acorus calamus*	*Xenopus* oocytes	α_1_β_2_γ_2S_	EC_5–10_	588 ± 126	300	65.3 ± 21.6	[87]
**193**	*Aristolochia manshuriensis*	*Xenopus* oocytes	α_1_β_2_γ_2S_	EC_5–10_	70.7 ± 2.6	1000	56.02 ± 5.09	[140]
**194**	*Atractylodes macrocephala*	*Xenopus* oocytes	α_1_β_2_γ_2S_	EC_1–10_	96 ± 3	300	12 ± 1	[141]
**195**	*Atractylodes macrocephala*	*Xenopus* oocytes	α_1_β_2_γ_2S_	EC_1–10_	166 ± 122	300	70 ± 17	[141]
**196**	*Atractylodes macrocephala*	*Xenopus* oocytes	α_1_β_2_γ_2S_	EC_1–10_	157 ± 12	300	99 ± 20	[141]
**198**	*Acremonium strictum*	*Xenopus* oocytes	α_1_β_1_γ_2S_	EC_20_	180	1	0.05 ± 0.02	[145]
**202**	*Boswellia serrata*	*Xenopus* oocytes	α_1_β_2_γ_2S_	EC_5–10_	397.5 ± 34.0	100	8.7 ± 1.3	[148]
**203**	*Platycladus orientalis*	*Xenopus* oocytes	α_1_β_2_γ_2S_	EC_5–10_	425.2 ± 96.5	500	141.6 ± 68.0	[149]
**204**	*Platycladus orientalis*	*Xenopus* oocytes	α_1_β_2_γ_2S_	EC_5–10_	855.7 ± 114.9	500	33.2 ± 8.7	[149]
**207**	*Curcuma kwangsiensis*	*Xenopus* oocytes	α_1_β_2_γ_2S_	EC_3–10_	309.4 ± 35.6	300	24.9 ± 8.8	[151]
**208**	*Curcuma kwangsiensis*	*Xenopus* oocytes	α_1_β_2_γ_2S_	EC_3–10_	211.0 ± 26.0	300	35.7 ± 8.8	[151]
**220**	*Panax ginseng*	*Xenopus* oocytes	α_1_β_2_γ_2S_	10 µM			53.2 ± 12.3	[162]
**221**	*Panax ginseng*	*Xenopus* oocytes	α_1_β_2_γ_2S_	10 µM	23.3 ± 1.4	100	23.1 ± 8.6	[163]
**222**	*Panax* *ginseng*	*Xenopus* oocytes	α_1_β_2_γ_2S_	10 µM	54.1 ± 1.7	100	17.1 ± 2.2	[163]
**228**	*Actaea racemosa*	*Xenopus* oocytes	α_1_β_2_γ_2S_	EC_3–10_	378 ± 64	300	36 ± 14	[167]
**229**	*Actaea racemosa*	*Xenopus* oocytes	α_1_β_2_γ_2S_	EC_3–10_	256 ± 40	300	28 ± 17	[167]
**230**	*Actaea racemosa*	*Xenopus* oocytes	α_1_β_2_γ_2S_	EC_3–10_	289 ± 45	300	26 ± 7	[167]
**231**	*Actaea racemosa*	*Xenopus* oocytes	α_1_β_2_γ_2S_	EC_3–10_	1947 ± 185	300	27 ± 8	[167]

**Table 3:** Data from radioligand binding assays.

Cmpd	Source	Binding Site	Ligand	IC_50_ [µM]	*K*_i_ [µM]	Stimulation [%]	c [µM]	Ref.
**4**	*Leonurus japonicus*	GABA/muscimol	[^3^H]gabazine	15,000				[24]
**19**	*Aconitum leucostomum*	GABA/muscimol	[^3^H]muscimol	7.06				[31]
**218**		GABA/muscimol	[^3^H]GABA		64 ± 5			[160]
**2**	*Colchicum szovitsii*	benzodiazepine	[^3^H]flunitrazepam			25% of 10 µM allopregnanolone	10	[22]
**3**	*Colchicum szovitsii*	benzodiazepine	[^3^H]flunitrazepam			25% of 10 µM allopregnanolone	10	[22]
**4**	*Leonurus japonicus*	benzodiazepine	[^3^H]flumazenil	123,000				[24]
**16**	*Oceanapia sp.*	benzodiazepine	[^3^H]diazepam			39	25	[30]
**17**	*Oceanapia sp.*	benzodiazepine	[^3^H]diazepam			32	25	[30]
**18**	*Oceanapia sp.*	benzodiazepine	[^3^H]diazepam			30	25	[30]
**34**	*Scuttelaria baicalensis*	benzodiazepine	[^3^H]flunitrazepam		7.81 ± 1.81			[42]
**35**	*Scuttelaria baicalensis*	benzodiazepine	[^3^H]flunitrazepam		4.20 ± 0.27			[42]
**36**	*Scuttelaria baicalensis*	benzodiazepine	[^3^H]flunitrazepam		0.21 ± 0.10			[42]
**37**	*Scuttelaria baicalensis*	benzodiazepine	[^3^H]flunitrazepam		0.56 ± 0.07			[42]
**38**	*Scuttelaria baicalensis*	benzodiazepine	[^3^H]flunitrazepam		0.027 ± 0.003			[42]
**39**	*Scuttelaria baicalensis*	benzodiazepine	[^3^H]flunitrazepam	6.42 ± 0.95				[41]
**40**	*Scuttelaria baicalensis*	benzodiazepine	[^3^H]flunitrazepam		0.0075 ± 0.004			[42]
**41**	*Scuttelaria baicalensis*	benzodiazepine	[^3^H]flunitrazepam	0.28 ± 0.076				[41]
**42**	*Scuttelaria baicalensis*	benzodiazepine	[^3^H]flunitrazepam		2.64 ± 0.36			[42]
**43**	*Scuttelaria baicalensis*	benzodiazepine	[^3^H]flunitrazepam		0.034 ± 0.001			[42]
**44**	*Scuttelaria baicalensis*	benzodiazepine	[^3^H]flunitrazepam		9.46 ± 1.45			[42]
**45**	*Scuttelaria baicalensis*	benzodiazepine	[^3^H]flunitrazepam	0.69 ± 0.12	0.64 ± 0.26			[41,42]
**46**	*Scuttelaria baicalensis*	benzodiazepine	[^3^H]flunitrazepam	1.26 ± 0.24	1.52 ± 0.13			[41,42]
**47**	*Scuttelaria baicalensis*	benzodiazepine	[^3^H]flunitrazepam	0.36 ± 0.095	0.20 ± 0.05			[41,42]
**48**	*Scuttelaria baicalensis*	benzodiazepine	[^3^H]flunitrazepam	0.008 ± 0.0002	0.0061 ± 0.0001			[41,42]
**49**	*Scuttelaria baicalensis*	benzodiazepine	[^3^H]flunitrazepam	0.31 ± 0.088				[41]
**50**	*Scuttelaria baicalensis*	benzodiazepine	[^3^H]flunitrazepam		0.0038 ± 0.005			[42]
**51**	*Scuttelaria baicalensis*	benzodiazepine	[^3^H]flunitrazepam		5.58 ± 0.02			[42]
**52**	*Scuttelaria baicalensis*	benzodiazepine	[^3^H]flunitrazepam	21.0 ± 1.79				[41]
**53**	*Scuttelaria baicalensis*	benzodiazepine	[^3^H]flunitrazepam	21.4 ± 1.42				[41]
**54**	*Scuttelaria baicalensis*	benzodiazepine	[^3^H]flunitrazepam	10.1 ± 1.68				[41]
**55**	*Scuttelaria baicalensis*	benzodiazepine	[^3^H]flunitrazepam	>100				[41]
**56**	*Scuttelaria baicalensis*	benzodiazepine	[^3^H]flunitrazepam	0.14 ± 0.01	0.89 ± 0.06			[41,42]
**57**	*Scuttelaria baicalensis*	benzodiazepine	[^3^H]flunitrazepam	22.6 ± 1.30				[41]
**58**	*Scuttelaria baicalensis*	benzodiazepine	[^3^H]flunitrazepam	12.5 ± 1.58				[41]
**59**	*Scuttelaria baicalensis*	benzodiazepine	[^3^H]flunitrazepam	1.27 ± 0.08				[41]
**60**	*Scuttelaria baicalensis*	benzodiazepine	[^3^H]flunitrazepam	6.80 ± 1.18				[41]
**61**	*Scuttelaria baicalensis*	benzodiazepine	[^3^H]flunitrazepam	32.8 ± 1.51				[41]
**62**	*Scuttelaria baicalensis*	benzodiazepine	[^3^H]flunitrazepam	45.7 ± 2.48				[41]
**63**	*Tanacetum parthenium*	benzodiazepine	[^3^H]flumazenil	12	9			[60]
**63**	*Salvia officinalis*	benzodiazepine	[^3^H]flumazenil	30 ± 4				[65]
**63**	*Rhus pyroides*	benzodiazepine	[^3^H]flumazenil	10.0 ± 1.18				[59]
**63**	*Matricaria recutita*	benzodiazepine	[^3^H]flunitrazepam		4			[58]
**66**	*Artemisia herba-alba*	benzodiazepine	[^3^H]flumazenil	8				[66]
**66**	*Salvia officinalis*	benzodiazepine	[^3^H]flumazenil	1.3 ± 0.2				[65]
**67**	*Salvia officinalis*	benzodiazepine	[^3^H]flumazenil	350 ± 37				[65]
**68**	*Salvia coerulea*	benzodiazepine	[^3^H]flunitrazepam		200			[67]
**68**	*Salvia coerulea*	benzodiazepine	[^3^H]zolpidem		20			[67]
**69**	*Artemisia herba-alba*	benzodiazepine	[^3^H]flumazenil	100				[66]
**70**	*Artemisia herba-alba*	benzodiazepine	[*methyl*-^3^H]DZP	1.3				[68]
**71**	*Artemisia herba-alba*	benzodiazepine	[*methyl*-^3^H]DZP	22.7				[68]
**78**	*Hypericum perforatum*	benzodiazepine	[^3^H]flunitrazepam	0.0149				[74]
**78**	*Searsia pyroides*	benzodiazepine	[^3^H]flunitrazepam		37			[59]
**79**	*Searsia pyroides*	benzodiazepine	[^3^H]flunitrazepam		28			[59]
**85**	*Mentha aquatica*	benzodiazepine	[^3^H]flumazenil	2600				[78]
**87**	*Scuttelaria baicalensis*	benzodiazepine	[^3^H]flunitrazepam	21.4 ± 1.32				[41]
**91**	*Glycyrrhiza glabra*	benzodiazepine	[^3^H]flumazenil		1.63			[80]
**98**		benzodiazepine	[^3^H]flunitrazepam		0.453			[84]
**121**	*Angelica dahurica*	benzodiazepine	[^3^H]diazepam	8.0 ± 0.8				[98]
**122**	*Angelica dahurica*	benzodiazepine	[^3^H]diazepam	0.36 ± 0.03				[98]
**123**	*Angelica dahurica*	benzodiazepine	[^3^H]diazepam	12 ± 3				[98]
**154**		benzodiazepine	[^3^H]flunitrazepam	74.44				[120]
**156**		benzodiazepine	[^3^H]flunitrazepam	15.69				[120]
**157**		benzodiazepine	[^3^H]flunitrazepam	90.02				[120]
**160**		benzodiazepine	[^3^H]flunitrazepam	130.9				[124]
**181**	*Cyperus rotundus*	benzodiazepine	[^3^H]flunitrazepam		105.0 ± 1.60			[134]
**186**	*Mentha aquatica*	benzodiazepine	[^3^H]flumazenil	190,000				[78]
**199**	*Salvia miltiorrhiza*	benzodiazepine	[^3^H]flunitrazepam	0.3				[146]
**200**	*Salvia officinalis*	benzodiazepine	[^3^H]flumazenil	7.2 ± 0.7				[65]
**201**	*Salvia officinalis*	benzodiazepine	[^3^H]flumazenil	0.8 ± 0.1				[65]
**205**	*Aloysia virgata*	benzodiazepine	[^3^H]flumazenil		111 ± 13			[150]
**206**	*Aloysia virgata*	benzodiazepine	[^3^H]flumazenil		56 ± 19			[150]
**2**	*Colchicum szovitsii*	TBPS/picrotoxin	[^35^S]TBPS			25% of 10 µM allopregnanolone	10	[22]
**3**	*Colchicum szovitsii*	TBPS/picrotoxin	[^35^S]TBPS			25% of 10 µM allopregnanolone	10	[22]
**7**	*Corydalis cava*	TBPS/picrotoxin	[^3^H]bicuculline			121 ± 2	0.1	[26]
**8**	*Corydalis cava*	TBPS/picrotoxin	[^3^H]bicuculline			149 ± 2	0.01	[26]
**9**	*Corydalis cava*	TBPS/picrotoxin	[^3^H]bicuculline			130 ± 3	0.1	[26]
**10**	*Corydalis cava*	TBPS/picrotoxin	[^3^H]bicuculline			146 ± 7	0.01	[26]
**11**	*Corydalis cava*	TBPS/picrotoxin	[^3^H]bicuculline			147 ± 3	0.1	[26]
**24**	*Cicuta virosa*	TBPS/picrotoxin	[^3^H]EBOB	0.54 ± 0.03				[37]
**25**	*Cicuta virosa*	TBPS/picrotoxin	[^3^H]EBOB	2.01 ± 0.09				[37]
**26**	*Cicuta virosa*	TBPS/picrotoxin	[^3^H]EBOB	1.15 ± 0.09				[37]
**27**	*Cicuta virosa*	TBPS/picrotoxin	[^3^H]EBOB	6.01 ± 0.29				[37]
**28**	*Cicuta virosa*	TBPS/picrotoxin	[^3^H]EBOB	7.87 ± 0.83				[37]
**65**	*Valeriana jatamansi*	TBPS/picrotoxin	[^35^S]TBPS		0.50 ± 0.17			[63]
**79**	*Rhus parviflora*	TBPS/picrotoxin	[^35^S]TBPS	0.149	0.091			[75]
**80**	*Rhus parviflora*	TBPS/picrotoxin	[^35^S]TBPS	0.073	0.045			[75]
**81**	*Rhus parviflora*	TBPS/picrotoxin	[^35^S]TBPS	0.455	0.280			[75]
**145**	*Ecklonia cava*	TBPS/picrotoxin	[^35^S]TBPS	7.180	4.419			[80]
**146**	*Ecklonia cava*	TBPS/picrotoxin	[^35^S]TBPS	1.739	1.070			[80]
**147**	*Ecklonia cava*	TBPS/picrotoxin	[^35^S]TBPS	4.991	3.072			[80]
**148**	*Ecklonia cava*	TBPS/picrotoxin	[^35^S]TBPS	2.422	1.491			[80]
**154**		TBPS/picrotoxin	[^35^S]TBPS	13 ± 4				[117]
**155**		TBPS/picrotoxin	[^35^S]TBPS	37 ± 8				[117]
**197**	*Illicium anisatum*	TBPS/picrotoxin	[^3^H]EBOB	0.43				[144]
**212**	*Ginkgo biloba*	TBPS/picrotoxin	[^35^S]TBPS	39				[154]

**Table 4:** Data from in vivo studies.

Cmpd	Anxiety/Stress										Sedation			Convulsions			Myorelaxation		Analgesia		Ref.
	VC	FP	EPM	OF	HB	LD	MB	FS	TS	SIH	LMA	PIS	EIS	MES	PTZ	PTX	RR	HW	TF	TI	
**11**			0.5		50													50			[27]
**12**								5													[28]
**13**								5													[28]
**14**								2.5													[28]
**15**			10	–								–					–				[29]
**19**	0.25																				[32]
**21**			+			+									+						[34]
**45**			1	–	3										+			–	–	25	[50,51,52,53]
**46**			7.5		7.5						–			5	5		–	–			[48,49]
**54**	10		+										+		–						[43,44]
**55**	10																				[43]
**56**			–		–						–						–	–			[47]
**63**			0.5	50	30						25		0.6			25		–			[55,58,61]
**65**			1	–																	[64]
**68**												2									[67]
**72**			0.2	–																	[69]
**73**			0.5																		[70]
**75**															50						[71]
**76**															100						[72]
**77**			2.5	5		5															[73]
**80**												12.5									[75]
**84**												5									[77]
**86**			1																		[64]
**98**												25									[84]
**103**			20									0.1									[89,90]
**105**												0.05									[92]
**107**			3																		[93]
**109**			4		4																[94]
**120**												5									[97]
**121**			5		5	5								300							[102,103]
**125**			5		5	5															[103]
**127**														259							[101]
**130**														150							[105]
**142**			50																		[111]
**143**			300	100	100							100									[112]
**144**											2.5			2.5	5						[113]
**149**												5			50						[116]
**158**														200	300	200					[121]
**159**															72.75						[122]
**163**			25		25			25	25												[125]
**171**																					[126]
**172**			25	25			25										75				[127,128]
**173**			25	25		25	25										–				[129]
**174**														400	100	200					[130]
**180**				100																	[107]
**182**			3																		[137]
**185**															10						[138]
**187**												+									[139]
**199**		10																–			[146]
**205**					0.3						1										[150]
**206**			1		0.3	1															[150]
**213**																			+		[157]
**214**			0.5															–			[156]
**215**																			+		[157]
**216**																			+		[158]
**217**				10								3									[159]
**219**			30																		[161]
**223**			10								50										[164]
**224**			10																		[164]
**225**			10																		[165]
**226**			10																		[165]
**231**			0.6	6						0.2					20						[168]
